# Evidence for phloem loading via the abaxial bundle sheath cells in maize leaves

**DOI:** 10.1093/plcell/koaa055

**Published:** 2021-01-07

**Authors:** Margaret Bezrutczyk, Nora R Zöllner, Colin P S Kruse, Thomas Hartwig, Tobias Lautwein, Karl Köhrer, Wolf B Frommer, Ji-Yun Kim

**Affiliations:** 1 Institute for Molecular Physiology, Heinrich-Heine-University Düsseldorf, Düsseldorf 40225, Germany; 2 Los Alamos National Laboratory, Los Alamos, New Mexico 87545, USA; 3 Biological and Medical Research Center (BMFZ), Genomics and Transcriptomics Laboratory (GTL), Medical Faculty, Heinrich Heine University Düsseldorf, Düsseldorf 40225, Germany; 4 Institute of Transformative Bio-Molecules (WPI-ITbM), Nagoya University, Chikusa, Nagoya 464-8601, Japan

## Abstract

Leaves are asymmetric, with different functions for adaxial and abaxial tissue. The bundle sheath (BS) of C_3_ barley (*Hordeum vulgare*) is dorsoventrally differentiated into three types of cells: adaxial structural, lateral S-type, and abaxial L-type BS cells. Based on plasmodesmatal connections between S-type cells and mestome sheath (parenchymatous cell layer below bundle sheath), S-type cells likely transfer assimilates toward the phloem. Here, we used single-cell RNA sequencing to investigate BS differentiation in C_4_ maize (*Zea mays L.*) plants. Abaxial BS (^ab^BS) cells of rank-2 intermediate veins specifically expressed three SWEET sucrose uniporters (SWEET13a, b, and c) and UmamiT amino acid efflux transporters. *SWEET13a*, *b*, *c* mRNAs were also detected in the phloem parenchyma (PP). We show that maize has acquired a mechanism for phloem loading in which ^ab^BS cells provide the main route for apoplasmic sucrose transfer toward the phloem. This putative route predominates in veins responsible for phloem loading (rank-2 intermediate), whereas rank-1 intermediate and major veins export sucrose from the PP adjacent to the sieve element companion cell complex, as in *Arabidopsis thaliana*. We surmise that ^ab^BS identity is subject to dorsoventral patterning and has components of PP identity. These observations provide insights into the unique transport-specific properties of ^ab^BS cells and support a modification to the canonical phloem loading pathway in maize.

## Teaser

Maize appears to use a previously undescribed phloem loading mechanism, in which abaxial bundle sheath cells export sucrose to transfer it to the vasculature.

## Introduction

Leaves are typically asymmetric; there are often differences in the relative stomatal and trichome densities and cuticle properties between the abaxial (lower) and adaxial (upper) leaf surfaces (Nelson et al., 2002; Juarez et al., 2004b). Although maize leaves are amphistomatic (stomata on both surfaces of the leaf), asymmetry remains apparent, with higher density of stomata on the abaxial side, bulliform cells only on the adaxial side and a conjoined, collateral and closed-type vasculature having adaxial xylem and abaxial phloem. Dorsoventral patterning in maize leaf is initiated in the shoot apical meristem at the earliest stages of leaf primordia development by expression of *Ragged seedling2* (*RGD2*; Henderson et al., 2006) and adaxial expression of *Rolled leaf1* (*RLD1*), which is conferred by miRNA166-mediated *RLD1* transcript cleavage on the abaxial side (Juarez et al., 2004a, 2004b). This pattern is maintained throughout development by specific localization of numerous transcription factors, including the abaxial expression of *KANADI* (Candela et al., 2008). Bundle sheath cells (BSCs) of maize are not known to be functionally differentiated. In barley leaves, the BSCs are anatomically distinct: abaxial side L-type BSCs have large chloroplasts, whereas S-type BSCs, with small chloroplasts, surround the rest of the mestome sheath. Because of the rapid disappearance of starch after the light period and the abundant plasmodesmatal connections between S-type cells, mestome sheath, and phloem, it was proposed that the S-type cells may be specialized for photoassimilate transport (Williams et al., 1989).

In maize, the two abaxial BS cells (^ab^BSCs) are typically smaller compared with the medial BSCs (Bosabalidis et al., 1994). In situ hybridization and immunolocalization had shown that Rubisco, the glutamine synthetase isoform GS1-4 (encoded by *GLN3*), and malic enzyme all localized specifically to BS, with transcripts and proteins equally represented in all BSCs (Langdale et al., 1988a; Martin et al., 2006). Here, we used single-cell RNA sequencing (scRNA-seq) to test whether the BS of the C_4_ plant maize is uniform or also has a dorsoventral differentiation of BSCs, as found in barley. Our analysis not only identified transcripts that were enriched in mesophyll (MS) and BS but also indicated that the BS could be subclustered into two groups, one expressing a variety of genes, including SWEET13 sucrose transporters, UmamiT, and AAP amino acid transporters, as well as several transcription factors. Results of in situ hybridization and analysis of translational β-glucuronidase (GUS) fusions demonstrated that the subclustering was due to a difference in expression along the dorsoventral axis, and this was evidenced by the finding that the three SWEET13 paralogs were specifically expressed in ^ab^BSCs. These findings not only show that the maize leaf BS is functionally differentiated but also indicate a previously undescribed route for apoplasmic phloem loading in a C_4_ plant. In addition, the three SWEET13 proteins were also present in cells that most likely represent the phloem parenchyma (PP) in a subset of veins, similar to the profiles of the phylogenetically related *Arabidopsis thaliana* (Arabidopsis, *At*) homologs At-SWEET11, 12, and 13. Maize ^ab^BS thus appear to have acquired aspects of PP identity and are differentiated from other BS cells along the dorsoventral axis, and are likely a key element of the phloem loading pathway in maize.

## Results

### mRNA patterns of specific cell types in maize leaves

**Figure 1:**
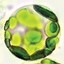
Mesophyll and bundle sheath clusters show canonical expression of C_4_ photosynthesis-related genes. A, Workflow for expression analysis in maize cells. Illustration of protoplasting, 10× chromium gel bead-in-emulsion (GEM) partitioning and cDNA synthesis, RNA sequencing, and data analysis. B, UMAP plot showing a two-dimensional representation of cell relationships from scRNA-seq data set A in multidimensional space. Bundle sheath cells separate into two subclusters at higher resolution (inset). The upper and lower clusters were later determined to correspond to abaxial and adaxial BS cells (Figure 2), and are therefore named ^ab^BS and ^ad^BS (C) Violin plots showing distribution of normalized mRNA counts of marker genes for cells in each cluster. Genes listed are known to be differentially expressed in MS and BS cells in C_4_ maize (*PEP1, MDH6, CA, ME1, RBCS1*) or are genes that identify unique clusters (*NAAT1, TAAT*). D, Violin plots showing normalized mRNA levels both of genes differentially expressed between ^ab^BS and BS^ad^ subclusters (*SWEET13a, SWEET13b, and SWEET13c*, *CC3 (cystatin3*) and of genes highly expressed in both clusters (*ME1*, *RBCS1*). Gene symbols are shown in Figure 1, C and D, and corresponding gene IDs from the most complete current B73 genome annotation available on the Ensembl genome browser (B73 RefGen_v4), as well as full names, are provided in Supplemental Table S2.

To determine whether maize leaves contain multiple BS cell types, we performed single-cell RNA sequencing on protoplasts isolated from maize leaves. We first established a protocol for protoplast release as described next. To minimize the possibility of a developmental gradient across cells, fully differentiated tissue was harvested from the distal portion of leaf 2 of late V2 stage plants (first and second leaf collar exposed; Li et al., 2010; Figure 1A). Standard leaf protoplasting protocols release individual MS cells and leave intact BS strands, consisting of BSCs and the vasculature (Kanai and Edwards, 1973; Langdale et al., 1989). In a parallel study, we were able to optimize protoplasting of Arabidopsis leaves to increase the yield of vascular cell types (Kim et al., 2021). We compared published protocols for releasing protoplasts from maize and varied parameters such as incubation time, enzyme concentration, enzyme blend, and preincubation in L-Cys (Ortiz-Ramírez et al., 2018). Efficiency of the release of putative vascular cells was monitored using qRT-PCR with cell type-specific markers (*PEPC*, *NADP-mDH* for MS, *NADP-ME* for BS and *SWEET13a*, *b*, and *c*; *18S rRNA*, *actin* as ubiquitously expressed genes), under the assumption that the *SWEET13* paralogs, analogous to their Arabidopsis homologs, would be specific to PP. Although none of the protocols were able to yield efficient release of putative vascular cells, we obtained the apparent release of both BSCs and vascular cells with Protocol 4, though the majority of cells released had MS characteristics (Supplemental Figure S1).

An estimated 7,000 protoplasts were pipetted together with reagents for cDNA synthesis into a 10× chromium microfluidic chip, where protoplasts were partitioned into bar coded gel beads (GEMs), together reagents for single-cell cDNA synthesis. Single-cell cDNA libraries were sequenced using next-generation sequencing (NGS). Gel bead-specific barcodes were used to identify mRNAs present in specific cells. After filtering this dataset (dataset A) to select for healthy cells, 3,763 cells with an average of 4,874 mRNA molecules per cell were analyzed. Unsupervised clustering was performed using Seurat (Butler et al., 2018) to determine the relationship between mRNA expression profiles , and this was ultimately represented in a two-dimensional Uniform Manifold Approximation and Projection plot (UMAP, a dimension reduction technique for visualization; Figure 1B). Cell identities were assigned to the clusters based on established marker genes for different cell types (Supplemental Table S1).

**Figure 2:**
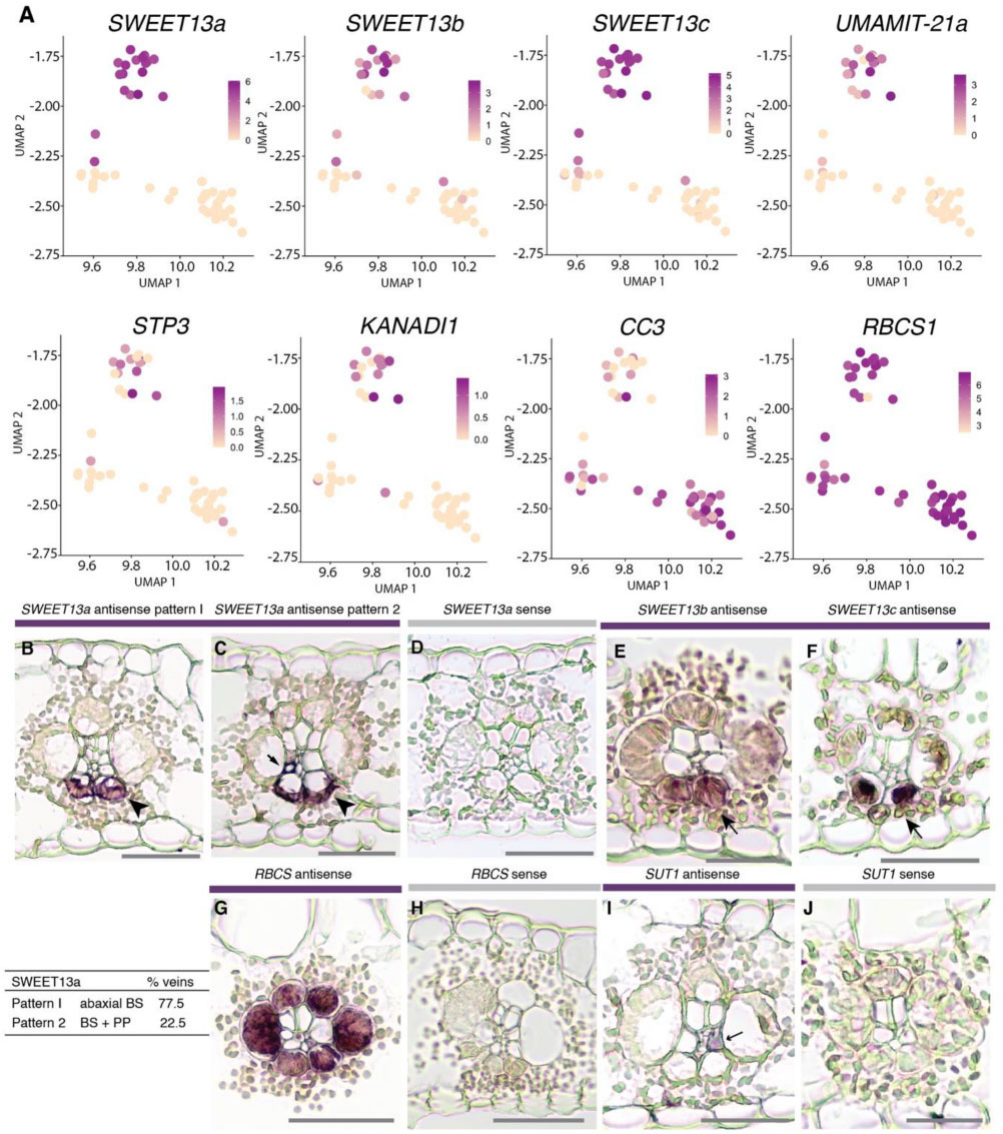
The abaxial BS cluster contains high levels of mRNAs encoding transport proteins. A, Feature plots show normalized levels of mRNAs for maize genes differentially expressed between the two clusters of bundle sheath cells from data set A plotted in UMAP space. B–F, In situ hybridizations of *SWEET13a*, *SWEET13b*, and *SWEET13c* to localize their mRNAs. Rank-2 intermediate veins from sections hybridized with antisense probes for *SWEET13a*, *SWEET13b*, and *SWEET13c* showed that the mRNA localization of three *SWEET13* genes was largely limited to abaxial bundle sheath cells. B and C, *SWEET13a* mRNA localization was predominantly in ^ab^BSCs in the majority of veins (77.5%) and to PP in a subset of veins (22.5%; *n* = 824). D, No staining was visible after hybridization with the *SWEET13a* sense probe (negative control). E and F, *SWEET13b* and *SWEET13c* probes showed staining predominantly in ^ab^BSCs. G, *RBCS1* antisense probe hybridized sections showed staining in all BS cells. H, No staining was detected in sections hybridized with an *RBCS1* sense probe. I, *SUT1* mRNA was localized to a vascular cell which is likely a companion cell in rank-2 intermediate veins (arrow). J, No staining was detected after hybridization with a *SUT1* sense probe (negative control). See Supplemental Figure S7 for intermediate rank-1 and major veins. Bars = 100 *µ*m.

A second independent scRNA-seq experiment yielded 3,242 cells. Data set A (from the above experiment) was analyzed in detail, the sparser data set B (from the second experiment), was used only for validation and comparison (Supplemental Figure S2). Analyzing data set A, we obtained six clusters, five of which formed a large supercluster that all had MS identities plus one separate cluster corresponding to BS identity (Figure 1C). The distribution of marker genes was consistent with the roles of MS and BS cells in C_4_ photosynthesis (Supplemental Figure S3 and Table S1). The ratio of MS cells to BSCs was approximately 75:1, indicating a low efficiency of BSC retrieval. To our surprise, no vascular cells were recovered. The BS cluster was further divided into two subclusters, the upper and lower subclusters in the UMAP plot, which later were assigned as abaxial (^ab^BS) and adaxial (^ad^BS) BS cells (Figures 1, D and 2; see below). Importantly, the separation of the BS cluster into subclusters which contained either high or low (or undetectable) levels of *SWEET13a, SWEET13b*, and *SWEET13c* mRNA, respectively, was robust when tested with multiple clustering parameters (Supplemental Figure S4).

### MS and BS clusters show canonical expression of C_4_ photosynthesis-related genes

Unsupervised clustering resulted in five clusters of MS cells based on the presence of genes involved in photosynthesis that are known to be differentially expressed between MS cells and BSCs (Friso et al., 2010; Li et al., 2010; Denton et al., 2017; Figure 1C; Supplemental Data Set 1 and Table S1). At first sight, the presence of five clusters was surprising. Marker genes and enriched genes are those which either are differentially expressed in a single cluster compared to all other clusters, or are differentially expressed between two specific clusters (as noted), and meet statistical significance criteria described in Supplemental Data Set 1. Cluster MS1 included most MS cells and likely represents the core MS. Cluster MS1 was enriched in photosynthetic processes. Cluster MS2 was enriched for the GO terms triose phosphate transport (GO:0035436; GO:0015717), nucleic acid metabolic process, immune system process (GO:0002376), and RNA metabolic process (GO:0016070) (Supplemental Data Sets 1 and 2). Clusters MS3 and MS4 contained high levels of ribosomal protein-related transcripts. Whether these ribosomal protein-enriched clusters represent biologically relevant cell populations in the leaf or are due to artifacts was not further evaluated due to our focus on BSCs. An additional subcluster, MS5, had an apparent MS identity, but was clearly separated from other MS clusters. The main determinants for this separate clustering were iron/metal-related processes. However, MS5 was not detected in data set B (Supplemental Figure S2). Because the M5 cluster was not reproducible, it was not analyzed further (Supplemental text). Our clustering data are supported by the presence of transcripts for the glutamine synthetase *GLN4* (corresponding to *gln1–3* and GS1–3 protein) in the MS1–4 cells (Supplemental Figure S5), consistent with previously published in situ and immunolocalization data that detected glutamine synthetase specifically in the MS (Martin et al., 2006).

It will be interesting to further explore whether the subclustering of MS cells represents developmental trajectories or physiological differences. We did not identify an obvious pattern that could be due to, for example, dorsoventral patterning due to developmental gradients or be due to changes in light properties as it passes through the leaf. Similar observations regarding the presence of multiple MS clusters were made for scRNA-seq analyses of Arabidopsis leaves; however, it was not possible to assign palisade and spongy parenchyma to any of the MS clusters (Kim et al., 2021).

### NADP-ME C_4_ photosynthesis-related transcripts are present in both BSC subsets

In maize, photosynthetic activity is partitioned between the MS and BSC (Li et al., 2010; Chang et al., 2012; Friso et al., 2010), allowing us to differentiate between these cell populations based on their mRNA profiles. Maize leaves utilize a combination of the NADP-ME- and PCK-dependent C_4_ pathways (Pick et al., 2011; Wingler et al., 1999), and MS cells and BSC must exchange metabolites via plasmodesmata, with specific enzymes highly upregulated in one cell type or the other. To identify the clusters, we selected several key marker genes that are known to be differentially expressed in either MS or bundle sheath.

Most of the genes involved in primary carbon metabolism that showed significant differential expression in the 2010 proteomics survey of MS and BS chloroplasts (Friso et al., 2010) were expressed in the expected cell types in dataset A (Supplemental Figure S3, Supplemental Data Set 1, and Supplemental Table S1). For example, transcripts of NADP-dependent malic enzyme (*ME1*) were almost exclusively found in BSCs, while carbonic anhydrase (*CAH1*) transcript levels were high in MS cells (Supplemental Figure S3, Supplemental Data Set 1, and Supplemental Table S1), in agreement with both proteomics data (Friso et al., 2010) and their corresponding contributions to C_4_ photosynthesis (Schlüter and Weber, 2020). All cells in the BSC subcluster showed high levels of mRNA for photosynthesis-related genes that function in the BSCs in NADP-ME/PCK C_4_ plants, including *RBCS*, *ME1*, and *PCK1* (Supplemental Figure S3, Supplemental Data Set 1, and Supplemental Table S1). The Rubisco small subunit mRNAs (*RBCS1*, *RBCS2*) were equally distributed in both abaxial and adaxial BS subclusters (Figure 1D; Supplemental Data Set 1). The bundle sheath-enriched glutamine synthetase *GLN3* (corresponding to gene *gln1-4* and protein GS1–4) was found in both BS subclusters, consistent with in situ and immunolocalization data that showed no evidence for a specific pattern for GS1–4 in the BS (Supplemental Figure S5; Martin et al., 2006).

### Abaxial BS cluster is enriched for genes encoding transport proteins

The BS cell subclusters (Figure 1D) could represent either developmental trajectories along the leaf axis (Li et al., 2010), BS cells from the three different vein classes (major vein or rank-l or rank-2 intermediate veins; Esau, 1943; Russell and Evert, 1985; Sharman, 1942; Hughes et al., 2019), different physiological states, or dorsoventral patterning. Whereas the majority of mRNAs corresponded to BS identity, only 5 genes were enriched in ^ad^BSCs (the lower subcluster) and 39 to ^ab^BSCs (the upper subcluster; Table 1). Surprisingly, among the genes with the largest difference in mRNA levels between the two BS subclusters were the three *SWEET13a*, *b*, and *c* paralogs. SWEETs are uniporters (Chen et al., 2010), and maize SWEET13a, b, and c function as sucrose transporters.

**Table 1:** Genes differentially expressed between clusters abBS and adBS

^ab^BS cluster	logFC	FDR	Description
Zm00001d023677	4.990	2.3E-08	**SWEET13a**
Zm00001d041067	3.959	3.2E-08	**SWEET13c**
Zm00001d016625	3.675	3.3E-07	Os02g0519800 protein
Zm00001d023673	2.156	2.2E-06	**SWEET13b**
Zm00001d035717	2.140	3.3E-07	**UmamiT21a**
Zm00001d033551	1.412	3.3E-07	Phosphoglycerate mutase-like family protein
Zm00001d033980	1.252	4.5E-03	*Ustilago maydis* induced12
Zm00001d019062	1.180	2.9E-04	**Membrane H^+^ATPase3**
Zm00001d035243	1.123	1.4E-03	**AAP45**
Zm00001d038753	1.114	7.7E-05	Ubiquitin domain-containing protein
Zm00001d017966	1.098	5.0E-04	Dihydropyrimidine dehydrogenase (NADP(+)) chloroplastic
Zm00001d012231	1.055	9.2E-04	**AAP56**
Zm00001d018867	0.992	1.1E-05	Syntaxin 132
Zm00001d000299	0.920	1.1E-05	Endosomal targeting BRO1-like domain-containing protein
Zm00001d002489	0.809	4.5E-03	PLATZ transcription factor family protein
Zm00001d035651	0.806	5.0E-04	DNA binding with one finger3
Zm00001d027268	0.753	6.5E-04	**STP3 (sugar transport protein 3)**
Zm00001d005344	0.737	1.4E-03	Histidine-containing phosphotransfer protein 2
Zm00001d013296	0.737	4.5E-03	ATP sulfurylase 1
Zm00001d030103	0.735	8.1E-03	Probable xyloglucan endotransglucosylase/hydrolase protein
Zm00001d052038	0.723	3.8E-03	Putative HLH DNA-binding domain superfamily protein
Zm00001d012559	0.692	2.1E-03	Stomatal closure-related actin-binding protein 1
Zm00001d015025	0.684	4.5E-03	AMP binding protein
Zm00001d038768	0.636	5.0E-03	Reticulon-like protein B4
Zm00001d044768	0.633	3.8E-03	**Protein NRT1/ PTR FAMILY 5.8**
Zm00001d033981	0.633	4.8E-03	ATP sulfurylase 1
Zm00001d015618	0.619	8.1E-03	Probable cinnamyl alcohol dehydrogenase
Zm00001d041710	0.597	5.0E-03	Glutathione synthetase chloroplastic
Zm00001d036401	0.596	5.0E-03	Endoplasmin homolog
Zm00001d018758	0.584	8.1E-03	Succinate dehydrogenase1
Zm00001d019670	0.578	8.5E-03	Kinesin-like protein KIN-4A
Zm00001d022042	0.574	9.4E-04	Eukaryotic translation initiation factor 5A
Zm00001d049597	0.567	4.9E-03	External alternative NAD(P)H-ubiquinone oxidoreductase B4
Zm00001d016662	0.536	3.8E-03	
Zm00001d018178	0.535	4.7E-03	bZIP4 (ABSCISIC ACID-INSENSITIVE 5-like protein 5)
Zm00001d032249	0.532	2.1E-03	KANADI 1
Zm00001d002625	0.531	3.4E-03	Probable methyltransferase PMT15
Zm00001d021773	0.517	9.9E-03	
Zm00001d039270	0.512	3.5E-03	Glutaredoxin family protein

The table shows the genes of interest that were differentially expressed between clusters ^ab^BS and ^ad^BS of data set A. Bold type = 9 out of 39 genes enriched in the ^ab^BS cluster that are specific to transmembrane transport. Criteria for inclusion were average log-fold change >0.5 for all cells in the subcluster and an FDR-adjusted *P* <0.01. The ^ab^BSC specificity was validated for three genes, *SWEET13a*, *b*, and *c*. Whether genes with lower FDR-adjusted *P*-values also show high specificity will require experimental validation. BS, bundle sheath; FC, fold-change.

**Figure 3:**
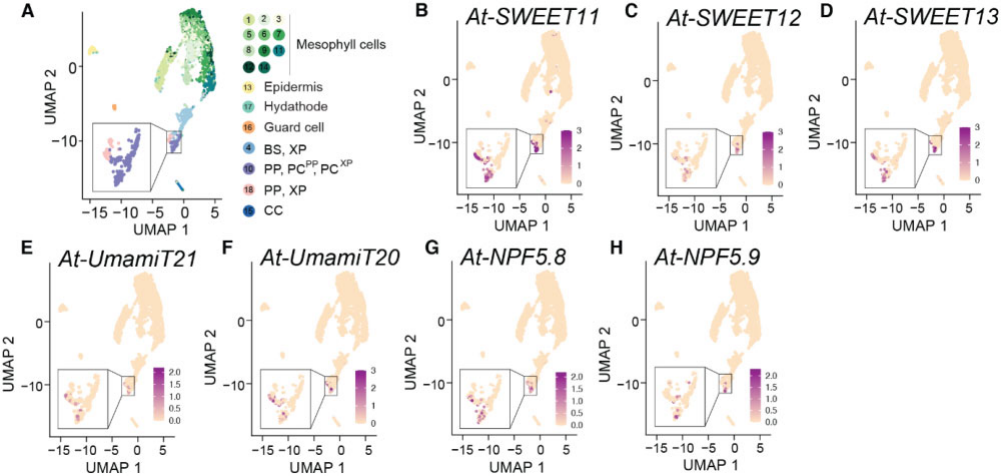
Arabidopsis homologs of genes enriched in maize ^ab^BS cells are expressed in Arabidopsis PP. Maize transporters showing mRNA enrichment in ^ab^BSCs are homologous to many Arabidopsis transporters enriched in Arabidopsis PP cells. A UMAP plot showing two-dimensional representation of cell relationships in multidimensional space for single-cell sequencing of Arabidopsis (At) leaf cells. Clusters are indicated by colors in the key to the right of the UMAP plot. Feature plots show normalized levels of mRNA transcripts for Arabidopsis transport proteins homologous to ^ab^BS transport proteins. B–D *At-SWEET11* (AT3G48740), *At-SWEET12* (AT5G23660), and *At-SWEET13* (AT5G50800) are homologous to *Zm-SWEET13a*, *Zm-SWEET13b*, and *Zm-SWEET13c*. E, *At-UmamiT21* (AT5G64700) is homologous to *Zm-UmamiT21a*. F, *At-UmamiT20* (AT4G08290) is homologous to *Zm-UmamiT20a* (Supplemental Figure S6). G and H, *At-NPF5.8* (AT5G14940)_*and At-NPF5.9* (AT3G01350) are homologous to *Zm-NRT1*.

Arabidopsis SWEET11 and SWEET12 are phylogenetically related to maize SWEET13a, SWEET13b, and SWEET13c, and in both species these SWEETs are critical for phloem loading of sucrose (Bezrutczyk et al., 2018). However, we did not know either if the maize SWEET13 proteins were co-expressed in the same cells or which cell types they function in. All three *SWEET13* paralogs were present in BSCs in maize, whereas their homologs in Arabidopsis, *At-SWEET11*, *At-SWEET12*, and *At-SWEET13*, are enriched in PP (Kim et al., 2021). This observation is consistent with the qRT-PCR results performed during the optimization of the protoplast protocol, which detected *SWEET13* transcripts, but no vascular cells, recovered by scRNA-seq. The upper BS subcluster, ^ab^BS, showed a striking enrichment for transport proteins, with 9 of 39 ^ab^BS-specific genes involved in transport (Table 1). Importantly, this included not only *SWEET13a*, *b*, and *c* but also an STP hexose transporter (STP3), the amino acid efflux transporter *UmamiTT21a* (Supplemental Figure S6, Supplemental Data Set 3), two members of the H^+^/amino acid symporter family, *AAP56* and *AAP45* (Deng, 2014), a member of the nitrate peptide transport family, and the H^+^-ATPase AHA3 (Table 1). Notably, in Arabidopsis, transcripts for the closest Arabidopsis homolog of *Zm-UmamiT21a*, *At-UmamiT21*, were enriched in PP (Figure 3) and co-expressed with *At-SWEET11* and *At-SWEET12* in Arabidopsis. On the basis of the presence of SWEETs, UmamiT21, and other ^ab^BS-enriched candidates, we speculate that the Arabidopsis transcription factors that are involved in PP identity have been recruited in maize to ^ab^BSCs to drive the unique set of genes expressed in the ^ab^BS. The transcription factor *DNA binding with one finger3* is implicated in the regulation of SWEET gene expression in rice (Wu et al., 2018) and shows ^ab^BS-preferential expression in this dataset. Two other transcription factors, *bZIP4* (*ABA-insensitive 5-like protein*) and *MYB25* (just below *p*-value cutoff), were enriched in the ^ab^BS. In contrast, ^ab^BS-enriched, *bZIP* and *MYB25* were not BS-specific, but were only sparsely expressed in MS cells (Supplemental Figure S5). One of the more highly expressed genes in the adaxial BS cluster was cystatin3 (CC3), the function of which is not known.

Other BSC-expressed genes such as *RBCS1* showed equal transcript distribution across all BS cells, excluding the possibility of an artifact, for example, a gradient of cells differing in unique molecular identifier (UMI) counts. This included *UmamiT20a*, which was BS-enriched but equally expressed across both BS subclusters. The lack of specificity of many genes for subsets of BSCs is consistent with published data from in situ hybridization and immunolocalization of *RBCS1* and glutamine synthetase, neither of which showed dorsoventral patterning (Langdale et al., 1988a; Martin et al., 2006; Langdale et al., 1988b ;Figure 2, G and H).

Because the cells partitioned were all collected from plants grown under the same conditions on the same day, we cannot exclude the possibility that transcriptional states could be influenced by particular conditions such as undetected pathogen infection or differences in growth conditions. Therefore, a second scRNA-seq experiment was generated (data set B). Data sets A and B were combined so that the transcriptional profiles of cells from both replicates could be directly compared. In both datasets, *SWEET13a*, *SWEET13b*, and *SWEET13c* were expressed in a subset of BS cells in both replicates along with genes encoding other transport proteins such as UmamiT21a and STP3, as well as the abaxial-localized transcription factor *KANADI1*. In both scRNA-seq datasets, *RBCS1* and other BS-specific C4 genes were highly expressed in all BS cells (Supplemental Figure S2). A major difference was the absence of cluster M5, indicating that M5 likely represented a subset of cells in a different physiological state (Supplemental text).

To test the hypothesis that the two BS subclusters represent spatially discrete BSC populations, in situ hybridization was used to localize the *SWEET13a, b*, and *c* mRNAs (Figure 2, B–F; Supplemental Figure S7). Notably, all three *SWEET13* transcripts were specifically detected in the two abaxial BSCs (^ab^BSCs) adjacent to the phloem. Additionally, *SWEET13a, b*, and *c* transcripts localized to the PP (Figure 2C; Supplemental Figure S7) similar to those of *At-SWEET11*, *At-SWEET12*, and *13* (Kim et al., 2021; Chen et al., 2012). Using three independent sets of probes, we discovered that transcripts of all three SWEET13s were almost exclusively found in the ^ab^BS of rank-2 intermediate veins, which are a special adaptation of C_4_ monocots (Langdale et al., 1988a) and serve as the main sites of phloem loading (Fritz et al., 1989, 1983). *SWEET13a* was detected in both ^ab^BS and PP cells in about 23% of the rank-2 intermediate veins. Thus, in the veins that are the main loading sites, sucrose efflux toward the SE/CC for phloem loading must occur predominantly from the ^ab^BS into the apoplasm toward the phloem, and only to a smaller extent by direct release at the SE/CC from PP. Rank-1 intermediate veins seemed to have a more balanced distribution of *SWEET13a* between the ^ab^BS and PP. In major veins, SWEET13 transcripts were also present in the medial vascular parenchyma, and the main path appeared to be through release from PP.

**Figure 4:**
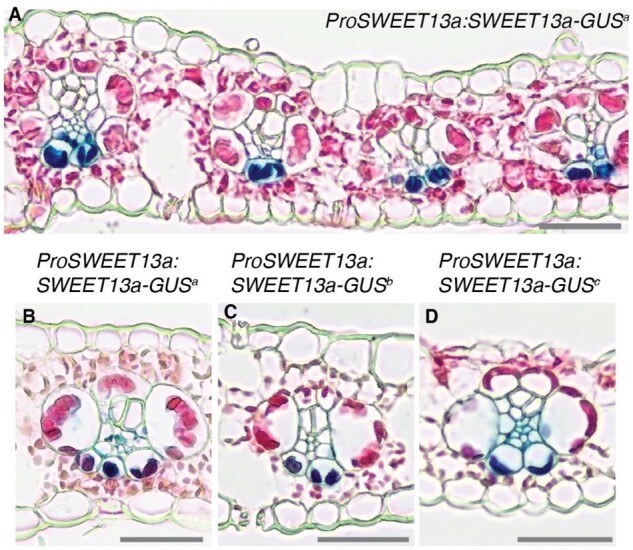
SWEET13a is localized to abaxial bundle sheath cells of rank-2 veins. GUS histochemistry in leaves of maize lines transformed with the translational fusion construct *ProSWEET13a:SWEET13a-GUS*. A, The dark blue chlorobromoindigo precipitate indicative of GUS activity was detected in abaxial bundle sheath cells of maize plants transformed with *ProSWEET13a:SWEET13a-GUSplus*. B–D, Three independent transformation events (a, b, and c = *ProSWEET13a:SWEET13a-GUSa*, *b*, and *c*) resulted in similar expression patterns in rank-2 intermediate veins, rank-1 intermediate veins, and major veins (for rank-1 and major veins see Supplemental Figure 7). B, Line “a,” (C) Line “b,” and (D) Line “c.” Sections were counterstained with eosin-Y; bars = 100 µm.

Because protein abundance does not always correlate with mRNA levels (Walley et al., 2016), we evaluated the cell specificity of the SWEET13a protein. Maize lines were generated that stably expressed translational GUS reporter fusions (*ProSWEET13a:SWEET13a-GUS*) comprising 6-kb upstream of the ATG and all exons and introns. Six transgenic lines from three independent transformation events showed consistent localization of the SWEET13a–GUS fusion protein in the ^ab^BS and PP of rank-1 and -2 intermediate veins, and in the PP of the major veins (Figure 4; Supplemental Figure S7).

In summary, both in situ hybridization and immunolocalization showed that *SWEET13a, b*, and *c* transcripts and SWEET13a protein were found not only in the PP of maize but also in a subset of BSCs, specifically the ^ab^BS. This is different from the cellular expression of their homologs in the dicot Arabidopsis, suggesting that an additional sucrose phloem loading route has evolved in C_4_ monocots (Langdale et al., 1988a).

### *SWEET13a*–*c* and *SUT1* sucrose transporters are expressed in complementary cell types

Sucrose released from cells by SWEETs is taken up actively into the SE/CC by SUT1 H^+^/sucrose symporters (Riesmeier et al., 1994; Bürkle et al., 1998; Gottwald et al., 2000; Slewinski et al., 2009). To compare SWEET13 and SUT localization directly, in situ hybridization was performed in parallel using the same method from leaves that were at the same stages of development. *SUT1* RNA was typically found in one or two phloem cells, which most likely represent companion cells, where it is responsible for phloem loading. In rank-1 and major veins, *SUT1* mRNA was also detected in the medial vascular parenchyma, where it likely contributes to sucrose retrieval (Heyser et al., 1977). In our experiments, *SUT1* transcripts were not detected in BSCs, consistent with *SUT1* expression below the detection limit in of our single-cell dataset (Figure 2, G–H; Supplemental Figure S7).

### Abaxial BS transcripts are co-regulated during the sink–source transition

**Figure 5:**
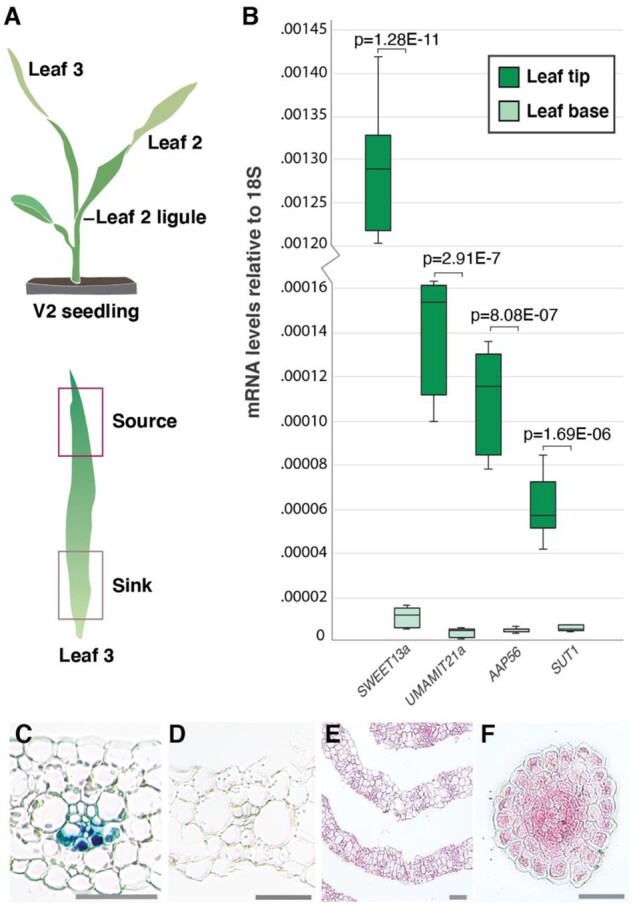
Abaxial BS transcripts are co-regulated during the sink–source transition. A, Tissues selected for qRT-PCR: a V2-stage seedling (upper) with source and sink tissue highlighted (lower). The base of leaf 3 is still in the whorl and is net sink tissue (Li et al., 2010). B, 18S-normalized mRNA levels of *SWEET13a*, *UmamiT21a*, *AAP45* (encoding proteins transport proteins enriched in ^ab^BS cells) and *SUT1* in source (leaf tip) and sink (leaf base) tissue. Values are average of three technical (qRT-PCR) replicates of three pools of two plants; error bars represent sem. *Students two-tailed paired *t* test values are shown. Independent repeats confirmed the data. C, *ProSWEET13a:SWEET13a-GUSplus* transformed B73 seedling segments after a 12–48 h incubation in GUS staining solution. V2 leaf 3 tip (12 h), (D) leaf 3 sheath (48 h), (E) stem cross section 1 cm above soil (48 h), and (F) cross-section across root tip (48 h). Of these, only the tip of leaf 3 (source) showed chlorobromoindigo precipitate indicative of GUS activity due to expression of the SWEET13a–GUS fusion protein. Bars: 100 *µ*m.

In Arabidopsis, many PP-expressed genes were found to also be co-regulated (Kim et al., 2021). We therefore tested whether several of the transporter genes identified in the ^ab^BS might also be co-regulated. *SUT1* H^+^/sucrose symporter genes are typically expressed to a low level in young net-importing leaves and are induced during the sink-to-source transition (Bürkle et al., 1998; Riesmeier et al., 1993). RNA was extracted from different segments of leaf 3 of V2 plants, in which the tip had transitioned to a sucrose source while the base was still in the sink state (Tausta et al., 2014; Figure 5A) for qRT-PCR. *SWEET13a*, Transcripts of *UmamiT21*, *AAP45*, and *SUT1* were 115-, 34-, 23-, and 10-fold higher, respectively, in the tip of leaf 3 (source) compared with the base (sink; Figure 5, A and B). SWEET13a protein levels were also higher in source regions of the leaf (Figure 5C). SWEET13a was detected neither in stem sections near the base of the plant, which contains whorls of developing leaves, nor in root tip (Figure 5, D–F). In leaves, SWEET13a was not detectable in tissues other than the tip of leaf 3, consistent with its role in phloem loading in source leaves. The co-regulation of ^ab^BS-enriched genes during the developmental transition of leaves not only links them to transfer of nutrients to the phloem but also indicates that they are all controlled by the same regulatory system.

## Discussion

Although single-cell RNA sequencing was successfully used to identify the transcriptomes of vascular cells in Arabidopsis (Kim et al., 2021), the suberin–lignin barrier surrounding the BS of maize leaves prevented access to vascular cell transcriptomes in maize. With our optimized protocol, BSCs were released and could be identified based on a broad range of known marker genes. BSCs separated into two subclusters. mRNA for BSC markers such as *RBCS* and GS1–4 were present at equal levels in both subclusters, whereas others were specifically enriched in one of the two subclusters. Because only moderately and highly expressed mRNAs are captured with droplet-based scRNA-seq protocols such as 10× chromium, we cannot exclude the possibility that transcripts that appear to be specific are also present at lower levels in the other cell types.

A major surprise was the finding that mRNAs for all three *SWEET13* paralogs were present in BSCs, in clear contrast to the distribution of their homologs in Arabidopsis (Chen et al., 2012). Other groups had shown that *SWEET13a*, *SWEET13b*, and *SWEET13c* transcripts are abundant in mechanically isolated BS strands, which likely also include vascular cells within the strands (see Supplementary discussion in Chang et al., 2012). Therefore, the data shown here provide multiple lines of evidence that *SWEET13a*, *SWEET13b*, and *SWEET13c* transcripts are found in specialized BSCs. Because barley BSCs seem clearly differentiated, with S-type cells suspected to represent a preferential site of sucrose transfer into the phloem (Williams et al., 1989), we tested whether *SWEET13a*, *b*, and *c* mRNA would be present in adaxial and lateral BSCs.

**Figure 6:**
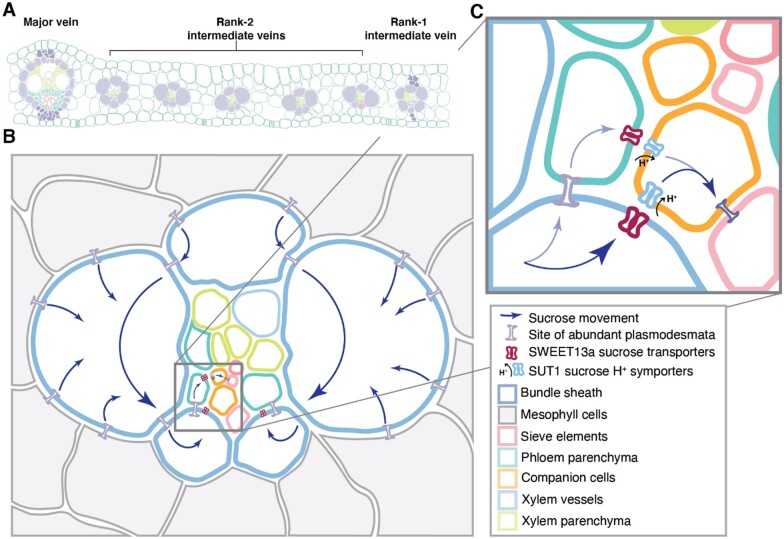
Phloem loading occurs via the ^ab^BS in maize. A, Arrangement and relative numbers of major veins, rank-1 intermediate veins, and rank-2 intermediate veins in a mature maize leaf. Note that rank-1 intermediate veins are distinguished from rank-2 by the presence of hypodermal sclerenchyma. B, A rank-2 intermediate vein surrounded by bundle sheath (blue outline) and mesophyll (gray) cells. Sucrose movement down its concentration gradient is indicated by blue arrows. C, The inset shows details of sucrose movement either from bundle sheath cells into the apoplasm via SWEET13 transporters or to PP (teal) via plasmodesmata, where sucrose is then exported to the apoplasmic space by SWEETs. Sucrose in the apoplasm is taken up by SUT1 into the sieve element (orange, pink) complex of the companion cells for long distance transport.

To our surprise, in situ hybridization and the analysis of translational GUS fusions showed that all three SWEET13s were preferentially expressed in the ^ab^BSCs of rank-2 intermediate veins, which are considered the main sites of phloem loading in maize (Fritz et al., 1983, 1989). In maize, these two abaxial BSCs are smaller compared with the medial BSCs (Bosabalidis et al., 1994). In rank-2 intermediate veins of maize, it appears that the ^ab^BSCs may have recruited sucrose-transporting *SWEETs* in order to export sucrose toward the abaxially localized phloem (Figure 6). Based on the amplification of *SWEET13* genes just prior to the evolution of C_4_ photosynthesis in both *Andropogoneae* and *Paniceae* lineages, Emms et al. (2016) hypothesized that SWEET13 transporters may export sucrose directly from the BS to the apoplasm in C_4_ plants. This conclusion was based on expression RNAseq data from BS strands (Chang et al., 2012) and laser capture dissection (Li et al., 2010), techniques that both cause contamination by adjacent tissues such as vascular parenchyma (see Supplementary materials and methods in Chang et al., 2012).

The presence of SWEET13s in the ^ab^BS possibly identifies a novel route for phloem loading in which BSCs likely export photosynthetically derived sucrose to the apoplasm of the phloem on the abaxial side of the leaf. Rank-2 veins are thought to be an emergent phenomenon of C_4_ grasses (Sedelnikova et al., 2018). Rank-2 veins increase the ratio of BS to MS cells, the vein density, and the capacity for nutrient transport, and they appear to be the main path for sucrose phloem loading. It is thus conceivable that this phloem loading route coevolved with the evolution of the rank-2 intermediate veins. A minority of rank-2 intermediate veins showed transcripts of *SWEET13a* in vascular cells, presumably PP, in addition to ^ab^BSCs, suggesting that, in a subset of rank-2 intermediate veins, sucrose may be exported from PP as well.

Given the findings of Williams et al. (1989), which indicate that barley uses adaxial and medial BSCs for phloem loading, our results suggest that the two species use distinct sets of BSCs for transferring sucrose from the BS to the phloem. This may occur in other C_3_ and C_4_ species, and it will be interesting to explore whether *SWEETs* are also present in medial BSCs of barley. ^ab^BS and ^ad^BS transcript profiles are highly similar, possibly explaining why this differentiation of BSCs had previously not been identified.

In rank-1 intermediate and major veins, *SWEET13a*, *b*, and *c* transcripts were detected in cells in the vasculature that most likely correspond to PP, thus similar to the canonical route in Arabidopsis (Chen et al., 2012; Cayla et al., 2019). Phylogenetic and functional analyses had shown that the PP transporters SWEET11 and SWEET12 from Arabidopsis are phylogenetically related to the three maize SWEET13 isoforms and fulfill overall a related function, that is, cellular sucrose efflux as a key step for phloem loading (Bezrutczyk et al., 2018). Subsequent to SWEET-mediated efflux, sucrose is taken up actively by SUT1/SUC2 H^+^/sucrose symporters in both maize and Arabidopsis (Gottwald et al., 2000; Slewinski et al., 2009). This is supported by in situ hybridization of *SUT1* in maize leaves (Supplemental Figure S7). The CC localization of *SUT1* is consistent with previously published results (Baker et al., 2016). We could not confirm previous data that indicated that SUT1 may also be expressed substantially in BSCs (Supplemental Figure S7). Localization of *SUT1* in vascular parenchyma is consistent with a role in sucrose retrieval on the side of the phloem that faces the xylem.

Our data are compatible with the presence of two distinct sites for phloem efflux in maize leaves, one from the ^ab^BS and a more standard path from PP in a minority of rank-2 veins (Figure 6). This route may be a specific adaptation of maize leaves in the context of C_4_ photosynthesis (Emms et al., 2016) to provide higher rates of sucrose flux toward the phloem. No doubt, *SWEET13a*, *b*, and *c* are key transporters for phloem loading, though at present we cannot assess the relative contribution of this new efflux step. This model could be tested by inhibiting SWEET activity specifically either in BSC or PP. However, because transcription factors driving expression of genes specifically in maize ^ab^BS or PP are not currently known, this hypothesis could be tested by generation of lines in which *SWEET13* mRNA levels have been repressed through BSC-specific RNAi.

Notably, transcripts for other transporter genes were also enriched in the ^ab^BS. This includes UmamiT21a, a member of the UmamiT amino acid transporter family. One of the key findings from the analysis of PP in Arabidopsis by scRNA-seq was that multiple members of this family were enriched in PP (Figure 3 and Table 1; Kim et al., 2021). Because they appear to play roles analogous to that of SWEETs in cellular efflux of amino acids in Arabidopsis, it appears that the ^ab^BS, besides having clear BSC identity, has acquired components or subnetworks of the PP identity. In both maize and Arabidopsis, BS and PP cell lineages are thought to diverge relatively early during vein development (Bosabalidis et al., 1994; Dengler et al., 1985; Esau, 1943), making it unlikely that ^ab^BS expression of SWEET13s is determined by cell lineage. Instead, positional cues may determine which cells adjacent to the SE/CC express the genes involved in nutrient transport. Mobile signals are known to be critical for patterning of root vasculature in Arabidopsis (De Rybel et al., 2016), and it is possible that positional cues, possibly originating in companion cells, contribute to the induction of transporter genes in adjacent cells.

The co-regulation of at least some of the ^ab^BS-enriched genes further strengthens this hypothesis. Interestingly, we also observed weak enrichment of the abaxial *KANADI* transcription factor (Figure 2A; Supplemental Data Set 1). KANADI plays a key role in determining abaxial identity in leaves (Candela et al., 2008). We therefore hypothesize that one or several transcriptional regulators that are involved in the regulation of the efflux of sucrose and amino acids from PP have been brought under control of both a polarity cue and the BS identity cues in order to increase nutrient flux toward the maize phloem. It will be fascinating to identify the transcription factors that are involved in controlling the PP and BSC identities and to dissect the SWEET promoters to determine which *cis*-elements are involved in the acquisition of ^ab^BSC fate. Several transcriptional regulators have been identified as candidates for the induction of ^ab^BSC fate; however, the limitations of sequencing depth in 10× genomics chromium droplet-based scRNA-seq preclude a comprehensive profiling of all transcriptional regulators. Methods that provide higher sensitivity may help to address this aspect. Importantly, the ^ab^BS genes identified provide unique insight into the specialized nature of this cell type.

Comparison of this phenomenon in other grasses, those that use both C_3_ and C_4_ photosynthesis, as well as a careful analysis of the evolution of rank-2 intermediate veins may provide insights into how widely distributed this mechanism is and may provide hints regarding the evolution of this regulatory rearrangement. Finally, new methods will be required to gain access to the vascular cells of maize, which are not accessible through the current methods. In summary, scRNA-seq enabled the identification of cells with a unique combination of properties on the adaxial side of the bundle sheath. The identification of this new property may be relevant to bioengineering of staple crops, for example, C_4_ rice.

### Material availability

Plasmids generated in this study have been deposited to Addgene under the code Plasmid #159535.

### Data and code availability

The raw data that support the findings of this study are available from the corresponding author upon request. All sequencing data have been deposited in the Gene Expression Omnibus (www.ncbi.nlm.nih.gov/geo/) under the accession number GSE157759.

## Materials and methods

### Plant growth

Maize (*Zea mays* L.) B73 seeds (obtained from Maize COOP) were germinated on filter paper with deionized, distilled water in darkness at 22–25°C and transferred to soil (Einheitserde ED73, Meyer KG, Frankfurt a.M., Germany) mixed with 20% perlite upon coleoptile emergence. Plants were subsequently grown at 28–30°C in a greenhouse supplemented by sodium lamps (400 *µ*mol m^−2^ s^−1^) for 12 h from 8:00 to 20:00. Protoplasts for scRNA-seq were generated from the last 6 cm of the distal portion of V2 leaf 2. For each pool of protoplasts tested, leaf segments from six concurrently grown plants were used. In situ hybridization was performed on sections taken from the distal portion of leaf 2 of V2 plants, the distal portion of leaf 5 of V4 plants, and the distal portion of the flag leaf from VT plants, with similar results. All images shown are from V4 leaf 5. For GUS staining, tissue segments were taken 10 cm from the tip of the third leaf below the flag leaf of T0 plants at growth stage VT (mature leaf tip), 4 cm from the tip of leaf 3 of T1 V2 plants (seedling leaf tip), 12 cm from the tip of T1 V2 plants (seedling leaf base), a stem section 1 cm above soil surface of T1 V2 plant (seedling stem), or the seminal root tip from T1 V2 plant (seedling root).

### Genes analyzed

Gene IDs are provided as Supplemental Table S2.

### Probe preparation for in situ hybridization

RNA was extracted from leaves of V2 B73 seedlings by a phenol–chloroform extraction method as previously described (Bezrutczyk et al., 2018). cDNA synthesis was performed using QuantiTect Reverse Transcription Kit (Qiagen, Hilden, Germany). cDNA was amplified for each gene (primers are listed in Supplemental Data Set 4) using Takara PrimeSTAR GXL polymerase then subcloned into pJET1.2 using CloneJet PCR cloning kit (ThermoFisher, Meerbusch, Germany). For *SWEET13a*, *b*, and *c*, three unique regions in the 5′- and 3′-UTRs and in the coding region with lengths of ∼100 bp were selected as probe templates. The three probes specific for one of the genes were combined for detection of the respective target gene. For *SUT1*, two regions in the 5′- and 3′-UTRs, unique to *SUT1* but common to all six isoforms, were selected as probe templates. For *RBCS1*, two regions in the coding region of the gene were selected as probe templates. All cDNA sequence alignments were performed using Geneious R11 (https://www.geneious.com; Supplemental Figure S8). Probe template regions were amplified with SP6 sequences flanking the forward primers for the sense probe, and reverse primers for antisense probes (Supplemental Data Set 4). Digoxigenin (DIG) labeled probes were generated using the MEGAscript SP6 Transcription kit (ThermoFisher) with a 1:2 ratio of DIG-labeled UTP:UTP. Probes were precipitated after the DNAse reaction by addition of 2 mg/mL glycogen, 0.1 volume 10% acetic acid, 0.1 volume NaOAc, and 2.5 volumes ethanol and were centrifuged at 4°C at 20,000*g* for 30 min. Pellets were washed with 70% ethanol in diethyl pyrocarbonate (DEPC)-treated water, allowed to dry, and resuspended in 25-*µ*L RNAse-free 10 mM Tris–EDTA pH 8 and 25-*µ*L formamide.

### In situ hybridization

In situ hybridization was adapted from Jackson and Simon lab protocols (Jackson, 1992; Stahl and Simon, 2010). Leaf-tip sections 1 cm in length were dissected from V2 or V5 plants and placed into 4% paraformaldehyde, vacuum infiltrated for 10 min, and fixed overnight at 4°C. Dehydration by ethanol series and paraplast embedding were performed as described (Malcomber and Kellogg, 2004). Sections (10 *µ*m) were cut with a Leica RM 2155 microtome and mounted on ProbeOn Plus slides (Fisher). After deparaffinization with Histoclear and rehydration by a decreasing ethanol series, tissue was permeabilized in a 2 *µ*g/mL proteinase K solution, washed for 2 min with 0.2% glycine and 1× PBS (1.3 M NaCl, 0.07 M Na_2_HPO_4_, 0.03 M NaH_2_PO_4_, pH), and fixed again with 4% paraformaldehyde for 10 min. Slides were washed with PBS and acetylated with 0.1 M triethanolamine and acetic anhydride for 10 min, then washed and dehydrated with an increasing ethanol series from 15% to 100%. Probes for each construct were mixed (e.g. all three antisense probes for *SWEET13a*), diluted 1:50 with formamide, denatured at 95°C for 3 min, and further diluted 1:4 with hybridization buffer (300 mM NaCl, 10 mM NaH_2_PO_4_, 10 mM Na_2_HPO_4_, 10 mM Tris–Cl pH 6.8, 5 mM EDTA, 50% formamide, 12.5% dextran sulfate, 1.25 mg/mL tRNA). Probe incubation in slide pairs was performed at 55°C overnight. Slides were rinsed three times with 0.2× SSC pH 7 (600 mM NaCl, 60 mM sodium citrate) at 55°C for 1 h, washed with block reagent solution (Roche), washed with BSA blocking solution (10 mg/mL bovine serum albumen, 0.1 M Tris–Cl, 150 mL NaCl, 0.3% Triton X-100) for 45 min, and incubated with anti-DIG AP-conjugated antibody (Roche, Lot # 16646822) for 2 h at 22°C. Slides were rinsed four times with BSA block solution for 15 min each, then in Buffer C (100 mM Tris pH 9.5, 50 mM MgCl_2_, 100 mM NaCl) for 15 min, and incubated with 50 *µ*L nitro blue tetrazolium chloride (NBT) and 37.5 *µ*L 5-bromo-4-chloro-3-indolyl-phosphate (BCIP) in 5 mL buffer C for 24–48 h. Slides were washed with water, dehydrated with an increasing ethanol series as noted above, and mounted with Eukitt Quick-hardening mounting medium. Images were taken with an Olympus CKX53 cell culture microscope equipped with an EP50 camera. In situ hybridization experiments for each gene/probe combination were performed as a minimum of three independent times.

### Quantification of mRNA in situ hybridization staining patterns in veins

Observations of hundreds of veins led us to determine that veins could be classified by the pattern of cell types stained as intermediate rank-2 type I (^ab^BS cells only) or type II (BS cells + vascular cells, presumably PP); intermediate rank-1 type I (^ab^BS + vascular cells) or type II (vascular cells only); major type I (abaxial PP only) or type II (abaxial and medial PP). Sections hybridized with antisense probes were harvested from two different leaves and observed under an Olympus CKX53 cell culture microscope, and veins corresponding to cell-type-specific expression in the different vein classes were counted.

### Generation of Zm-SWEET13-GUS constructs

*Zm-SWEET13a* (Supplemental Table S2), including 5,751-bp upstream of the start codon and 684-bp downstream of the stop codon, was isolated from B73 gDNA (Supplemental Data Set 4) and inserted into pJET using the CloneJET PCR cloning kit. The final construct consists of GUSplus inserted directly upstream of the *Zm-SWEET13a* stop codon, preceded by a 9-Ala linker, in the Golden Gate vector pGGBb-AG, the in silico cloning of which was performed using Geneious R11 (Supplemental Figure S8). The assembly of all fragments with the vector pGGBb-AG was performed using the Takara InFusion HD cloning kit, and validated by Sanger sequencing.

### Maize transformation

*Agrobacterium tumefaciens* strain EHA105 was transformed with the *Zm-SWEET13a:GUS* vector at the Crop Genome Engineering Facility at VIB Ghent (Vlaams Instituut voor Biotechnologie; https://www.psb.ugent.be/cgef). Transformed EHA105 cells carrying the respective plasmids were used to transform the inbred maize line B104 via Agrobacterium-mediated transformation of 600 immature embryos according to previously described methods (Coussens et al., 2012). In brief, callus formation was induced using auxins and transgenic cells selected over several weeks using phosphinotricin selection. Plantlets were then regenerated on hormone-free medium, and presence of the transgene confirmed using TraitCheck (Romer Labs; Butzbach, Germany) and PCR analysis. Three independent transformation events were derived from different starting immature embryos, yielding six plants in total: three plants from event A (*ProSWEET13a:SWEET13a-GUSplus^a^*), two from event B (*ProSWEET13a:SWEET13a-GUSplus^b^*), and one from event C (*ProSWEET13a:SWEET13a-GUSplus^c^*).

### GUS histochemistry

Tissue segments were taken 10 cm from the tip of the third leaf below the flag leaf of T0 plants at growth stage VT (mature leaf tip), 4 cm from the tip of leaf 3 of T1 V2 plants (seedling leaf tip), 12 cm from the tip of T1 V2 plants (seedling leaf base), on the stem 1 cm above the soil surface of T1 V2 plant (seedling stem), or from the seminal root tip of T1 V2 plant (seedling root) at hour 13:00. Tissue segments were dissected into cold acetone and vacuum infiltrated for 2 min, then vacuum infiltrated with GUS wash buffer (20 mM EDTA, 40 mM C_6_N_6_FeK_3_, 40 mM C_6_FeK_4_N_6_, 20% methanol, 57.6 mM Na_2_HPO_4_, 42 mM NaH_2_PO_4_, 0.1% Triton X-100), and incubated with GUS wash buffer including 0.2% X-Gluc at 37°C for 1–48 h. GUS activity was detected by production of a dark blue chlorobromoindigo precipitate by cleavage and subsequent oxidation from X-Gluc. Sections were dehydrated in 20%, 30%, and 50% ethanol for 30 min, fixed in formalin-alcohol-acetic acid (FAA: 50% ethanol, 3.7% formaldehyde, 5% acetic acid) for 30 min, and further dehydrated in 75% and 100% ethanol. Embedding was performed by incubating sections at 60°C in tert-butyl ethanol:paraplast dilutions at 3:1, 1:1, and 1:3 ratios. Melted Paraplast (100%) was changed twice daily for 3 days. Paraplast-embedded tissue was poured into blocks, and 10-*µ*m sections were cut with a Leica RM 2155 microtome. Sections were mounted on SUPERFROST PLUS Gold Slides (Thermo Scientific), deparaffinized with Histoclear, and mounted with Eukitt Quick-hardening mounting medium. This GUS staining procedure was repeated three times in both T0 and T1 generations with similar results.

### Single-cell sequencing: protoplast preparation

Tissue was sampled from the distal portion of leaf 2 (from 1 to 7 cm, as measured from the tip) from V2 plants. This region was selected because it is thought to be nonexpanding, nondifferentiating source tissue based on results from the RNAseq-defined developmental transcriptome of the maize leaf (Li et al., 2010). For each pool of protoplasts, leaf segments from six concurrently grown plants were used. Leaf segments were harvested at hour 9:00, and tape was applied to the adaxial epidermis to stabilize the tissue, which was scored every 5 mm from the midvein to leaf edge with a razor manifold consisting of scalpel blades taped together to ensure minimum distance between scores. Taped sections were placed abaxial-side down in pretreatment solution (2 mM l-Cys, 164 mM sorbitol) and vacuum infiltrated for 10 min with 2 min of active pumping. Taped sections were incubated with gentle agitation (30 r.p.m., IKA Rocker 3D orbital shaker) for an additional 20 min in pretreatment solution, then transferred to enzyme solution (cellulase Onozuka RS 1.25%, cellulase Onozuka R-10 1.25%, pectolyase Y-23 0.4%, macerozyme R-10 0.42%, sorbitol 0.4 M, MES 20 mM, KCl 20 mM, CaCl_2_ 10 mM, BSA 0.1%, ß-mercaptoethanol 0.06%) for 3.5 h with gentle agitation (30 r.p.m. on an orbital shaker). Protoplasts were carefully pipetted onto a prewetted Corning 70-*µ*m nylon mesh strainer placed in a tilted 50 mL Falcon tube to remove large debris, and were then slowly pipetted into a round bottom tube using a wide-bore 1 mL pipette. Protoplasts in the round-bottomed tube were centrifuged for 1.5 min at 100*g* in a Hettich Rotina 38R centrifuge. The enzyme solution was gently removed and replaced with cold wash solution (sorbitol 0.4 M, MES 20 mM, KCl 20 mM, CaCl_2_ 10 mM, BSA 0.1%). Protoplasts were carefully resuspended in wash solution and centrifuged for 30 s at 100*g*, then strained through a 70-*µ*m Scienceware Flowmi Cell Strainer to remove large debris. Washing solution steps were repeated four additional times to remove chloroplasts and small debris. Cell viability and concentration were quantified under an Olympus CKX53 cell culture microscope: 1 *µ*L 0.4% Trypan blue was added to 9 µL of resuspended protoplasts in wash solution and pipetted into the chamber of a C-Chip Neubauer Improved Disposable Haemocytometer (NanoEntek; Seoul, South Korea); healthy (unstained) cells were counted. Protoplasts were resuspended to a concentration of 1,200 cells/*µ*L. A variety of approaches to degrade the suberin–lignin-containing BSC walls with the addition of other enzymes failed to produce healthy cells (data not shown): Laccase (Sigma) and manganese peroxidase (Sigma), as well as enzymes provided by Novozymes (Copenhagen, Denmark), namely a cutinase, a fungal carbohydrase blend produced in *Aspergillus aculeatus*, a fungal β-glucanase blend produced in *Humicola insolens*, a pectinase preparation produced in *Aspergillus*, a xylanase blend, and a multienzyme complex containing carbohydrases, including arabinase, cellulase, β-glucanase, hemicellulose, and xylanase, were each added to the existing protocol to a final concentration of 1%–2% active enzyme weight/volume. Visual inspection of protoplasts during isolation revealed that addition of these enzymes caused protoplasts to rupture.

### Generation of scRNA-seq data set B

To generate data set B, tissues from six plants at the same developmental stage as in the first experiment (B73, V2, leaf 2 tip) were grown at a different time under the same growth conditions and were harvested for protoplast release. 10× Genomics chromium cell partitioning and sequencing were performed as described for the first replicate: percent.pt <4 and percent.mt <0.75 and nFeature_RNA >1,800 and nFeature_RNA <7,000; 3,242 cells passed this threshold. Normalization, scaling, and variable feature detection were performed using SCTransform (Hafemeister and Satija, 2019a). See Quantification and statistical analysis of Materials and methods section for further analysis.

### Protoplast protocol optimization

Several variations of the above protocol were tested prior to the final protoplast preparation, and the presence of diverse cell types was verified by qRT-PCR using primers specific to *MDH6*, *ME1*, *SWEET13a*, *SWEET13b*, and *SWEET13c* (Supplemental Data Set 4). Briefly, RNA was extracted from protoplasts using the RNEasy Mini Kit, and first-strand cDNA was synthesized using a Quantitect reverse transcription kit (Qiagen, Hilden, Germany). Quantitative reverse transcription PCR (qRT-PCR) was performed using Roche LightCycler 480 SYBR Green I Master polymerase on a Stratagene Mx3000P, and relative expression of transporter genes was calculated relative to *18S* rRNA and *Actin* using the 2^−ΔCT^ method. Modifications to the standard protoplast isolation protocol of 2 h in enzyme solution (Protocol 1, see previous section) included doubling the concentration of enzymes in solution (Protocol 2), isolating BS strands released after 2 h followed by continued incubation of filtered BS strands in fresh enzyme solution to deplete MS cells (Protocol 3), and incubating the leaf tissue in pretreatment solution (Protocol 4, 2 mM L-Cys and 164 mM sorbitol). The protocol that yielded the highest ratio of BS:MS marker genes (*ME1*:*MDH6*) included a pretreatment incubation step (164 mM sorbitol, 2 mM L-cys in MilliQ water) and 1× enzyme solution (Supplemental Figure S1).

### Cell partitioning, library preparation, and NGS

To aim for partitioning of 7,000 cells with the expectation that 3,500 cells would be sequenced, 6 *µ*L of the protoplast suspension with an estimated 1,200 cells/*µ*L was applied to the 10× genomics chromium microfluidic chip (Chemistry V3.0). Thereafter the standard manufacturer’s protocol was followed. Twelve cycles were used for cDNA amplification, and the completed cDNA library was quantified using an Agilent AATI Fragment Analyzer. Sequencing was performed at Novogene (Sacramento, CA, USA) on a single lane with the Hi-Seq platform and the standard PE150 sequencing parameters.

### Generation of single-cell expression matrices

Cellranger count (10× Genomics) was used to process fastq files provided by Novogene, with read 1 trimmed to 26 bp (r1-length = 26), as the first 26 bp of a 10× library R1 comprise the cell barcode and UMI index, and the remaining part contains the poly-A tail with no further information. A formatted reference genome was generated using Cellranger mkref using the maize B73 RefGen 4 (Jiao et al., 2017) whole genome sequence and annotation (fasta and gff3 downloaded from Ensembl B73 RefGen V4), to which reads were aligned using STAR (Dobin et al., 2013). For analysis of single-cell sequencing data, see Quantification and statistical analysis section.

### Phylogeny of UmamiT transporters

BLAST results from the Arabidopsis seed sequence At-UmamiT12 to maize (AGP v4 of the MaizeSequence database (Jiao et al., 2017) and barley (IBSC v2 from the International Barley Genome Sequencing Consortium; Mascher et al., 2017) were combined with BioMart (Smedley et al., 2009) results and filtered for the WAT1-related protein domain (panther ID PTHR31218). Genes passing this filter were selected as UmamiT family candidate genes. Two trees were generated: one using an alignment of all known splice variants, and one with only the representative transcript, with similar results. Alignments were performed in MEGA7 (Kumar et al., 2016) using MUSCLE with the following parameters: gap open penalty −2.9, gap extend penalty 0, hydrophobicity multiplier 1.2, max iterations 8, clustering method UPGMA for iteration 1, 2; UPGMB for all subsequent iterations, and lambda 24. The maximum likelihood tree was created from these alignments using IQTREE webserver (Trifinopoulos et al., 2016) using the BLOSUM62 substitution model and 1,000 bootstraps (Supplemental Data Set 3).

### Quantification and statistical analysis

#### Sample selection for scRNA-seq, qRT-PCR, and RNAseq

Plants chosen for protoplast release, qPCR, and RNAseq were randomly selected from among a larger number of individuals that had been grown concurrently and were at the same growth stage. True biological replicates (i.e. independently grown plants) were used as replicates for statistical analyses. The number of plants per sample and number of replicates is given in the respective figure legends or in specific sections of Methods. To ensure reproducibility, the plants used in successive experiments were grown in the same greenhouse under controlled conditions. Samples for a repeat of a given experiment were taken at the same developmental stage at the same time of day.

### Dimensionality reduction and cell clustering

The Seurat R package (v3.1; Butler et al., 2018) was used for dimensionality reduction analysis and dataset filtering. To remove cells with low mRNA count (nFeature_RNA) and doublets, as well as damaged cells with high chloroplast (pt) or mitochondria (mt) genome-derived transcripts, cells were filtered (percent.pt <4 and percent.mt <0.75 and nFeature_RNA >1,800 and nFeature_RNA <7,000). Normalization, scaling, and variable feature detection were performed using SCTransform (Hafemeister and Satija, 2019a). Cells were clustered using FindNeighbors to create a *K*-nearest neighbors graph using the first 50 principle components. FindClusters was used to iteratively group cells using a resolution of 0.2 or 23. These clusters were used as input for nonlinear dimensional reduction using UMAP (McInnes et al., 2018). This allowed us to visualize the relatedness of cells in the single-cell dataset based on their transcriptomic profiles, as represented by their positions in 2D space.

### Differential gene expression analysis across clusters

Genes differentially expressed across clusters or subclusters were identified by comparing the average normalized mRNA counts in cells of a given cluster to that of cells in all other clusters using the Seurat function FindMarkers. Genes with a false discovery rate (FDR) corrected *P* <0.05 and an average log-fold change (logFC) >0.5 were considered marker genes.

### Identification of cluster identities

Photosynthesis-related genes known to be differentially expressed between MSCs and BSCs in NADP-ME/PCK C_4_ plants were used as markers to define MSC and BSC clusters (Denton et al., 2017; Li et al., 2010; Schlüter and Weber, 2020; Friso et al., 2010 ;Supplemental Table S1). The cluster identified as BSCs was subdivided into two subclusters when FindClusters was applied with a resolution of 23, and differential gene expression analysis was performed on these two subclusters with FindMarkers (for subclusters: logFC >0.5, FDR ≤0.01; Supplemental Data Set 1)

### Integration of scRNA-seq data sets A and B

The two scRNA-seq replicates were combined using the integration function in Seurat. First, the same parameters were used for filtering for both datasets: to remove cells with low mRNA count (nFeature_RNA) and doublets, as well as damaged cells with high chloroplast (pt) or mt genome-derived transcripts, cells were filtered (percent.pt <4 and percent.mt <0.75 and nFeature_RNA >1,800 and nFeature_RNA <7,000). Each dataset was separately scaled and normalized using SCTransform (Butler et al., 2018; Hafemeister and Satija, 2019b). Integration was performed using nfeatures = 1,000, and all features (genes) were used to find anchors. UMAP plots and feature plots were generated as previously described. After cell selection, the integrated dataset consisted of 7,005 cells (3,763 from data set A and 3,242 from data set B), with four MS clusters and one BS cluster at low resolution (FindClusters resolution = 0.2) and two BS clusters at high resolution (FindClusters resolution = 26). The integrated dataset was used to produce Supplemental Figure S2 only. All other analyses were performed using data set A.

### qRT-PCR of transporter genes in seedling leaves

Leaf segments were harvested from the distal and proximal end (tip and base) of leaf 3 of early V2 plants at hour 13:00. Tissue was ground in liquid nitrogen and RNA was extracted as previously described (Bezrutczyk et al., 2018). First-strand cDNA was synthesized using Quantitect reverse transcription kit (Qiagen). qRT-PCR to determine relative mRNA levels was performed using a Stratagene Mx3000P with primers for *18S, Actin, SWEET13a*, *13b*, *13c*, *SUT1*, *UmamiT21a*, and *STP3* (Supplemental Data Set 4). Relative expression of transporter genes was calculated relative to *18S* and *Actin* using the 2^−ΔCT^ method for quantification, with similar results. Values shown in Figure 5B are the average of three technical (qRT-PCR) replicates of three pools of two plants each; error bars represent sem. Students two-tailed paired *t* test values are shown. Two independent repeats confirmed the data.

**Figure:**
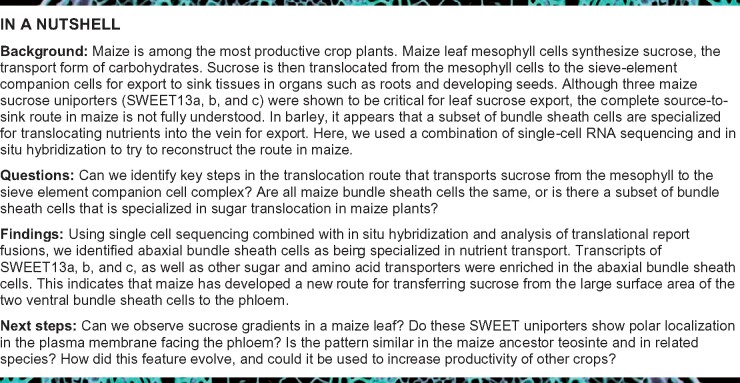


### Gene ontology term analysis for MS clusters

Marker genes for each of the five MS clusters (LogFC >0.5; FDR-adjusted *p*-value <0.05) were used as input for Gene Ontology (GO; Ashburner et al., 2000; The Gene Ontology Consortium, 2018) analysis via the online portal GO Gene Ontology database (doi: 10.5281/zenodo.3727280; released 2020-03-23). GO terms with false discovery rate (FDR)-corrected *P* <0.05 can be found in Supplemental Data Set 2.

### Protoplast and bulk leaf tissue RNAseq

Protoplasts were generated according to the previously described method. Whole leaf tissue from sibling plants was ground in liquid nitrogen at the time leaf tissue was harvested for protoplast isolation. RNA from two pools of protoplasts made from four leaves each (P1 and P2), and two pools of four whole leaf segments each (L1 and L2) was extracted using the RNEasy Mini Kit (Qiagen), and four cDNA libraries were generated using the NEBNext Ultra DNA Library Prep Kit for Illumina (New England Biolabs, MA, USA) with modifications to select for 250–500 bp fragments. Sequencing of the four libraries was performed at Novogene (Sacramento, CA, USA) on a single lane with the Hi-Seq platform and the standard PE150 sequencing parameters. Reads were analyzed using a custom implementation of the Wyseq RNAseq analysis pipeline (https://github.com/astauff/WySeq). Briefly, reads were trimmed using TrimGalore (v 0.6.5) and aligned to the AGPv4 B73 reference genome using STAR (v 2.5.1b). Counts were generated using Subread featureCounts (v 2.0.1), and differential expression was analyzed using the R-packages EdgeR (v3.30.3) and limma (v 3.44.3) using trimmed mean *M*-value normalization factors. Reads corresponding to genes enriched in either BS or MS cells were normalized separately to compensate for the expected difference in cell populations represented in protoplasts and whole leaf tissue. Genes were filtered to remove those with a coefficient of variation >75th percentile within replicate groups prior to correlation analysis. Pearson correlation (Supplemental Figure S9) and differentially expressed genes enriched in BS and MS cells (logFC > 1 or < −1) are presented (Supplemental Data Set 5). None of the genes in the ^ab^BS subclusters were induced by protoplast isolation. Rather, several showed reduced mRNA levels in the protoplast sample.

### Accession numbers

Accession numbers for maize genes are provided in Table 1; accession numbers Arabidopsis are provided in Supplemental Table 2. Additional genes used in this study include: actin—Zm00001d010159 and 18S rRNA—ENSRNA049479027.

## Supplemental data

**Supplemental Figure S1.** qRT-PCR of putative BSC and vascular-expressed genes as an indication of protoplast cell type diversity prior to sequencing.

**Supplemental Figure S2.** UMAP and feature plots of integrated datasets.

**Supplemental Figure S3.** Schematic of C4 photosynthesis-related genes and relative expression in BS and MS clusters.

**Supplemental Figure S4.** BS subclusters are robust to different clustering parameters.

**Supplemental Figure S5.** UMAP plots of glutamine synthetase, transport-related proteins, and transcription factors in bundle sheath cells.

**Supplemental Figure S6.** Neighbor joining tree of family of UmamiT amino acid transporters in Arabidopsis, maize, and barley.

**Supplemental Figure S7.** SWEET and SUT mRNA localization and SWEET13a protein localization in Rank-2 intermediate and major veins.

**Supplemental Figure S8.** Probe design for in situ hybridization and ProSWEET13a:SWEET13a-GUS construct schematic.

**Supplemental Figure S9.** Correlation of mRNA counts between protoplasted cells and whole leaf.

**Supplemental Table S1.** mRNA enrichment of C_4_ photosynthesis-related genes in MS and BS clusters.

**Supplemental Table S2.** Genes referenced in this study.

**Supplemental Text.**

**Supplemental References.**

**Supplemental Data Set 1.** Cluster-specific marker genes and degs across all clusters.

**Supplemental Data Set 2.** GO term analysis for MS clusters.

**Supplemental Data Set 3.** Raw data for phylogenetic trees.

**Supplemental Data Set 4.** Primers used in this study.

**Supplemental Data Set 5.** Protoplast-enriched and protoplast-depleted mRNAs

## Supplementary Material

koaa055_Supplementary_Data

## Notes

Conceptualization, M.B., J.K., W.B.F.; methodology, M.B., C.P.S.K, T.H., T.L.; investigation, M.B, N.Z, T.L, C.P.S.K.; resources, K.K, W.B.F.; writing, M.B., W.B.F.; and supervision, J.K., W.B.F.

The author(s) responsible for distribution of materials integral to the findings presented in this article in accordance with the policy described in the Instructions for Authors (https://academic.oup.com/plcell/pages/General-Instructions) is: Wolf B. Frommer (frommew@hhu.de).
