# Supplementary material for: Evidence for phloem loading via the abaxial bundle sheath cells in maize leaves
Source: Plant Cell. 2021 Jan 7;33(3):531–47. doi: 10.1093/plcell/koaa055 (PMC8136869; doi:10.1093/plcell/koaa055)
Supplement: koaa055_Supplementary_Data [file koaa055_supplementary_data.zip › tpc.00733.2020-s02.pdf]

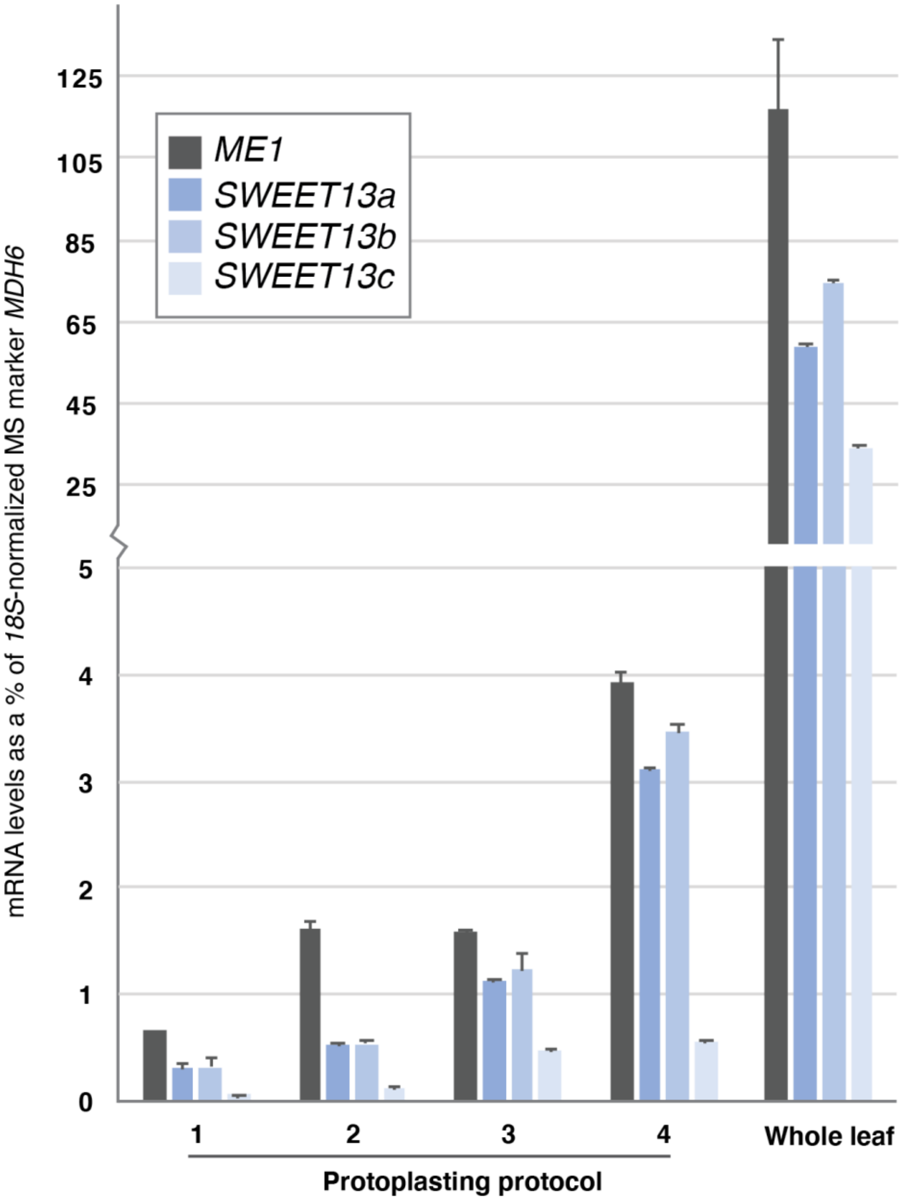

Supplemental Figure 1. qRT-PCR of Putative BSC and Vascular-Expressed Genes as an Indication of Protoplast Cell Type Diversity prior to Sequencing (Supports Figure 1).

Normalized mRNA levels of *ME1*, *SWEET13a*, *SWEET13b*, and *SWEET13c* shown as a percentage of *18S*-normalized expression of a mesophyll marker gene, *NADP-malate dehydrogenase6* (*MDH6*), after different protoplasting treatments. Error bars represent SEM of technical duplicates.

Protocol 1, standard enzyme cocktail (see Methods) with 3.5 h incubation. Protocol 2, doubled enzyme concentration. Protocol 3, isolated BS strands released after 2 h and continued protoplasting of filtered BS strands in fresh enzyme solution to deplete mesophyll cells. Protocol 4, incubated leaf tissue in pretreatment solution (2 mM L-cysteine and 164 mM sorbitol), which yielded the highest ratio of BS:MS marker genes.

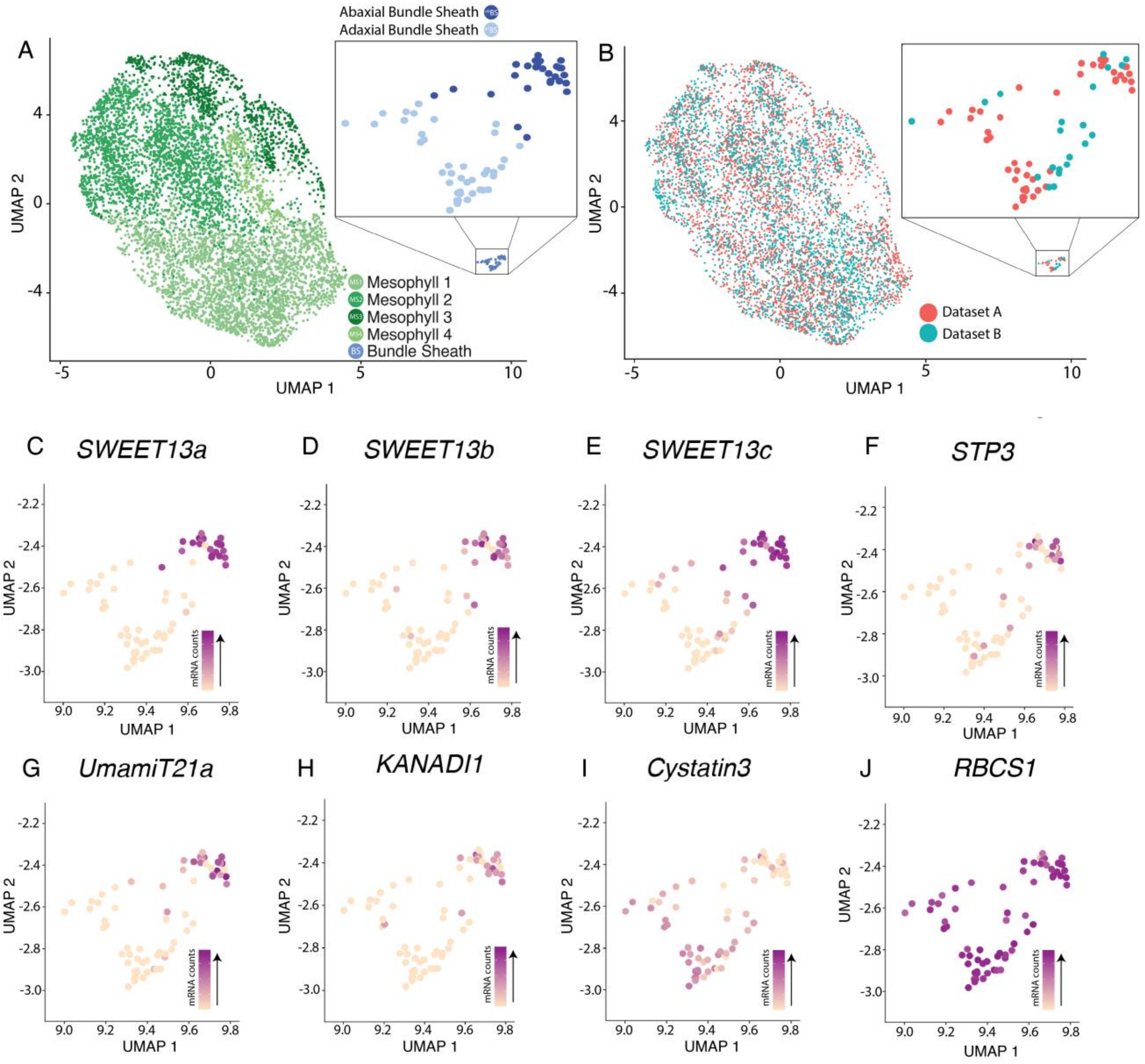

Supplemental Figure 2. UMAP and Feature Plots of Integrated Datasets (Supports Figure 1).

(A) The integrated dataset from the two independent replicates with cells colored by identity had four mesophyll clusters and one BS cluster at low resolution and two BS clusters at high resolution (inset panels) **(B)** UMAP plot with cells colored by dataset shows that clusters from different experiments can be superimposed on each other based on shared cell states/identities. UMAP plots of BS subclusters in the integrated dataset. **(C)** to **(J)** Feature plots show normalized levels of mRNAs plotted in UMAP space. *SWEET13a*, *SWEET13b*, *SWEET13c*, *UmamiT21a, STP3,* and *KANADI1* are all highly expressed in the abaxial BS cluster. *Cystatin3* is one of the only genes expressed preferentially in the adaxial BS cluster. *RBCS1* and other canonical C4 marker genes (not shown) are expressed in both clusters.

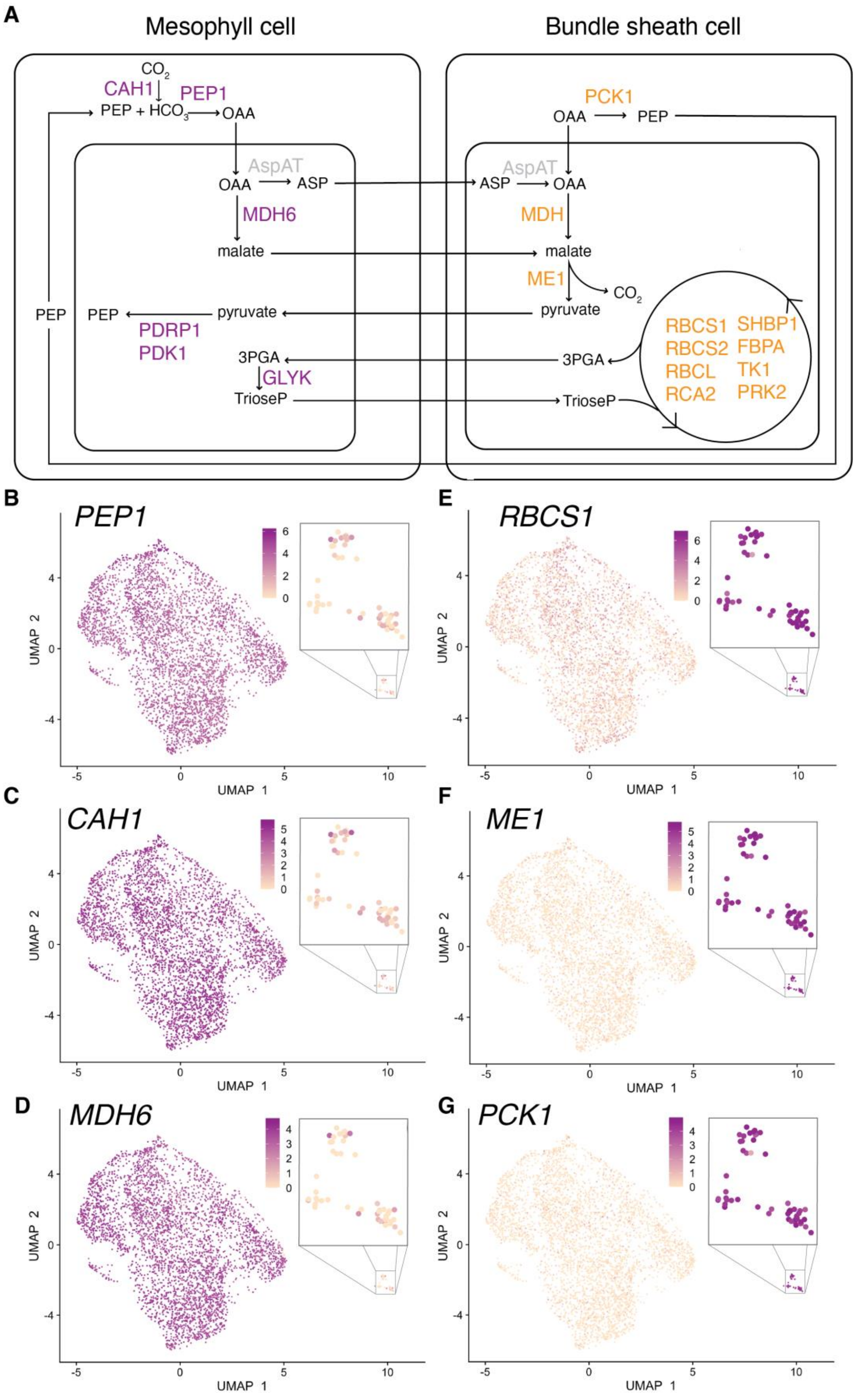
A
Mesophyll cell
Bundle sheath cell
CO2
CAH1
PEP1
PEP + HCO3
OAA
AspAT
ASP
MDH6
malate
PEP
pyruvate
PDRP1
PDK1
3PGA
GLYK
TrioseP
PCK1
MDH
ME1
RBCS1
RBCS2
RBCL
RCA2
SHBP1
FBPA
TK1
PRK2
B
PEP1
C
CAH1
D
MDH6
E
RBCS1
F
ME1
G
PCK1
UMAP 1
UMAP 2

Supplemental Figure 3. Schematic of C_4 Photosynthesis-Related Genes and Relative Expression in BS and MS Clusters (Supports Figure 1).

(A) Partitioning of proteins involved in C_4 photosynthesis between mesophyll and bundle sheath cells in maize (Schlüter and Weber, 2020; Friso et al., 2010). For all proteins displayed, mRNA levels in scRNA-seq dataset A were significantly enriched in either mesophyll or bundle sheath cells (logFC > 1.0; FDR-adjusted p-value < .05). Gene IDs, symbols, and full names are shown along with Log FC values in Supplemental Table 1. Genes which are shown but not differentially expressed indicated in grey: AspAT (Asp aminotransferase) (**B-G).** Feature plots show normalized levels of mRNAs for photosynthesis-related genes expressed differentially in mesophyll and bundle sheath. **(B)** *PEP1* (Phospho*enol*pyruvate carboxylase 1) **(C)** *CAH1* (Carbonic anhydrase 1) **(D)** *MDH6* (NADP-dependent malate dehydrogenase 6) **(E)** *RBCS1* (Ribulose bisphosphate carboxylase small subunit 1) **(F)** *ME1* (NADP-dependent malic enzyme 1) **(G)** *PCK1* (Phospho*enol*pyruvate carboxykinase 1).

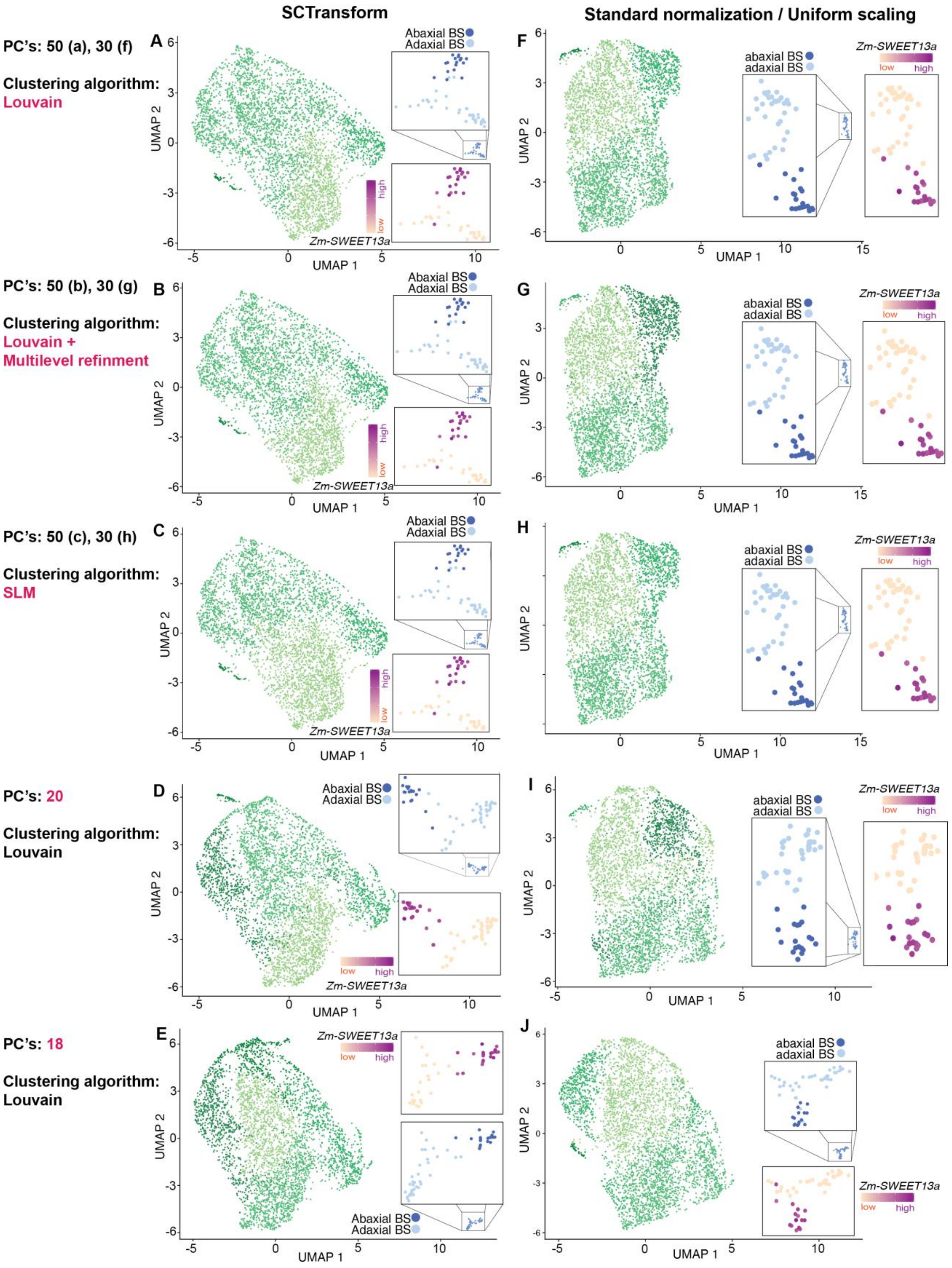
SCTransform
Standard normalization / Uniform scaling
PC's: 50 (a), 30 (f)
Clustering algorithm: Louvain
PC's: 50 (b), 30 (g)
Clustering algorithm: Louvain + Multilevel refinment
PC's: 50 (c), 30 (h)
Clustering algorithm: SLM
PC's: 20
Clustering algorithm: Louvain
PC's: 18
Clustering algorithm: Louvain
A
B
C
D
E
F
G
H
I
J
Abaxial BS
Adaxial BS
abaxial BS
adaxial BS
Zm-SWEET13a
low
high
UMAP 1
UMAP 2

Supplemental Figure 4. BS Subclusters Are Robust to Different Clustering Parameters (Supports Figure 1).

Clustering of single-cell RNAseq dataset A was performed with ten different unsupervised clustering parameters: three different modularity optimization algorithms **(A), (D), (E), (F), (I), and (J)** Louvain, **(B)** and **(G)** Louvain with multilevel refinement, and **(C)** and **(H)** SLM, two scaling and normalization methods **(A)** to **(E)** SCTransform and **(F)** to **(J)** Uniform scaling, as well as different numbers of principle components selected for KNN graph embedding **(E)** and **(J).** 18, **(D)** and **(I)** 20, and **(A)** to **(C), (F)** to **(H).** either 30 or 50, depending on the normalization and scaling method). In ten UMAP plots with corresponding feature plots showing normalized mRNA levels of SWEET13a, sub-clustering of the bundle sheath cells (inset panel) consistently subclustered two groups of cells, one of which was characterized by enriched levels of SWEET13a mRNA (inset panel), indicating that the BS subclusters presented in Fig 1a are robust to different clustering, normalization, and scaling parameters.

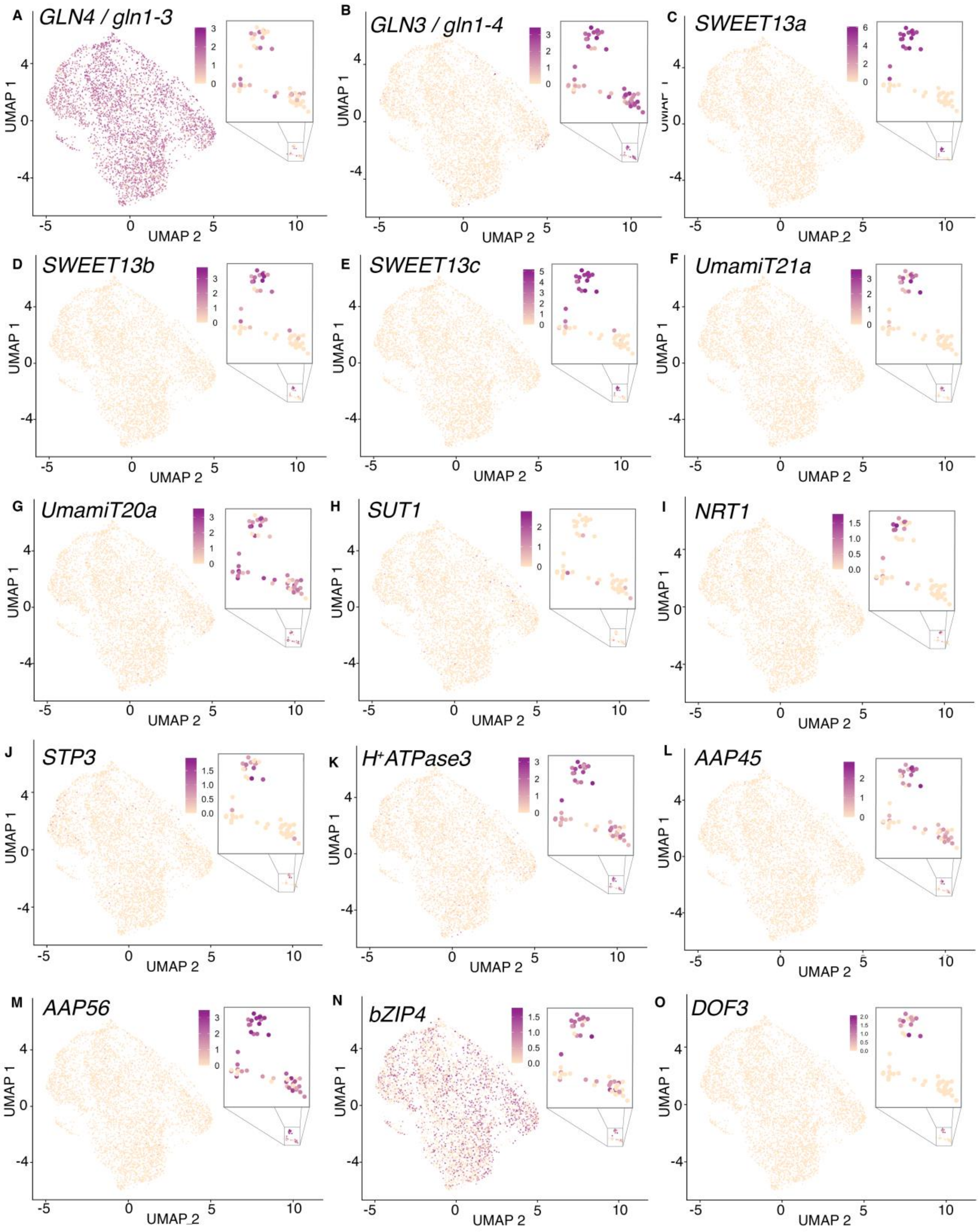
A GLN4 / gln1-3
B GLN3 / gln1-4
C SWEET13a
D SWEET13b
E SWEET13c
F UmamiT21a
G UmamiT20a
H SUT1
I NRT1
J STP3
K H+ATPase3
L AAP45
M AAP56
N bZIP4
O DOF3
UMAP 1
UMAP 2

Supplemental Figure 5. UMAP Plots of Glutamine Synthetase, Transport-Related Proteins, and Transcription Factors in Bundle Sheath Cells (Supports Figure 1).

(A) and **(B)** Feature plots show normalized levels of mRNA transcripts from scRNA-seq dataset A for glutamine synthetase genes expressed differentially in mesophyll and bundle sheath. **(A)** *GLN4 (gln1-3*, protein GS3, Zm00001d017958) is widely expressed in mesophyll cells and some bundle sheath cells. **(B)** *GLN3 (gln1-4*, protein GS4, Zm00001d028260) is expressed in bundle sheath cells. **(C)** to **(O)** Feature plots show normalized levels of mRNA transcripts for transport-related genes and transcription factors expressed in ^{ad}BS or ^{ab}BS. **(C)** *SWEET13a* (Zm00001d023677), **(D)** *SWEET13b* (Zm00001d023673), **(E)** *SWEET13c* (Zm00001d041067), and **(F)** *UmamiT21a* (Zm00001d035717) are enriched in ^{ab}BS. **(G)** *UmamiT20a* (Zm00001d044951) is expressed in ^{ab}BS and ^{ad}BS. **(H)** *SUT1* (Zm00001d027854) is not highly expressed in any cell type in this dataset. **(I)** *NRT1* (Zm00001d044768), **(J)** *STP3* (Zm00001d027268), **(K)** *H^{+}ATPase3* (Zm00001d019062), **(L)** *AAP45* (Zm00001d035243), **(M)** *AAP56* (Zm00001d012231), **(N)** *bZIP4* (Zm00001d018178) and **(O)** *DOF3* (Zm00001d035651) are enriched in ^{ab}BS relative to ^{ad}BS.

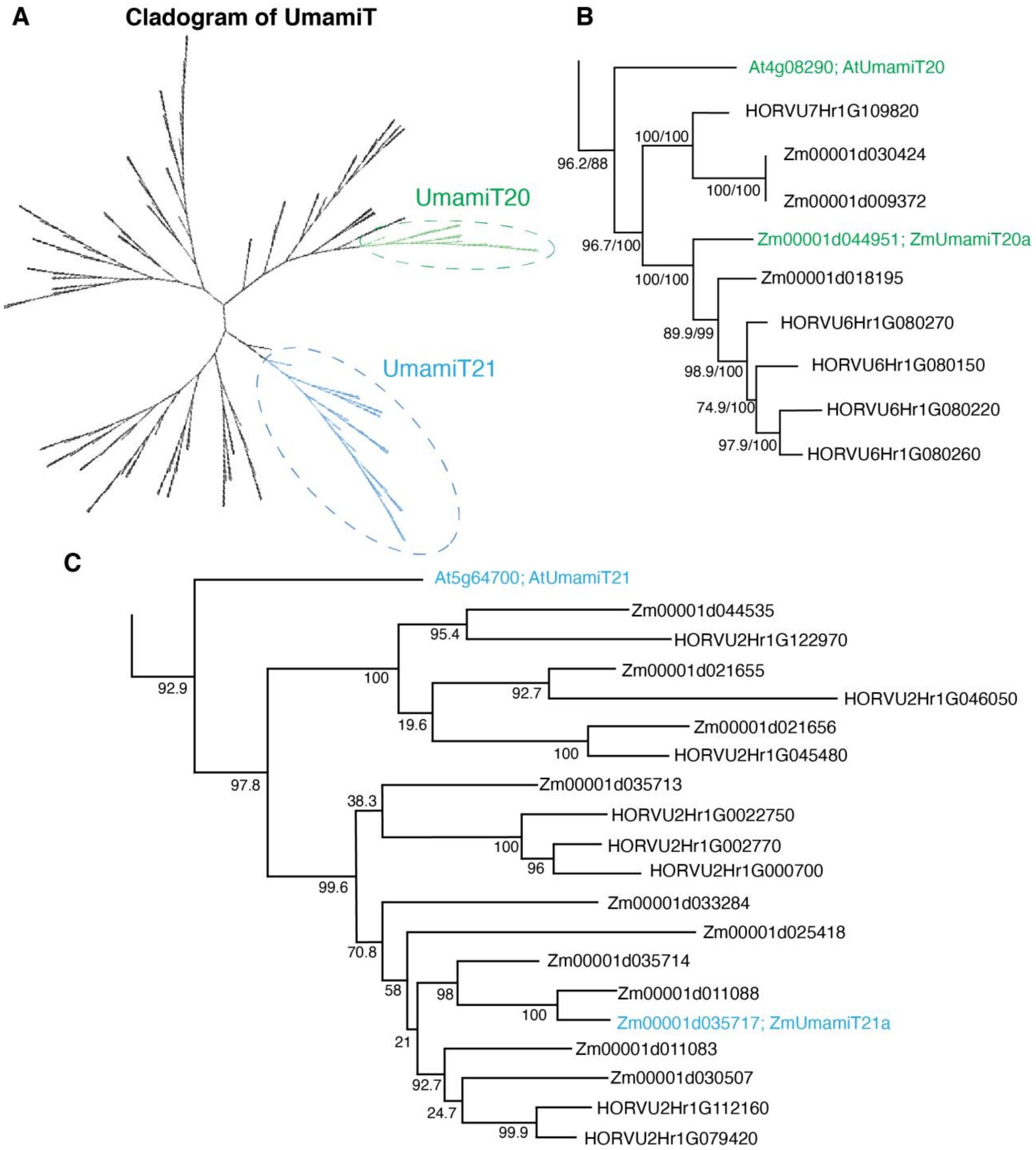

Supplemental Figure 6. Neighbor Joining Tree of Family of UmamiT Amino Acid Transporters in Arabidopsis, Maize, and Barley (Supports Figure 2).

(A) Cladogram of all UmamiT amino acid sequences in maize, barley, and Arabidopsis. **(B)** Phylogram of green highlighted clade containing the ZmUmamiT expressed in all BSC types. Zm00001d44951 is most closely related to Arabidopsis UmamiT20. **(C)** Phylogram of blue highlighted clade containing the ZmUmamiT enriched in ^{ab}BSC. Zm00001d035717 is most closely related to Arabidopsis UmamiT21. Amino acid sequences are indicated by the encoding gene ID. Values at nodes are % UF-bootstraps.

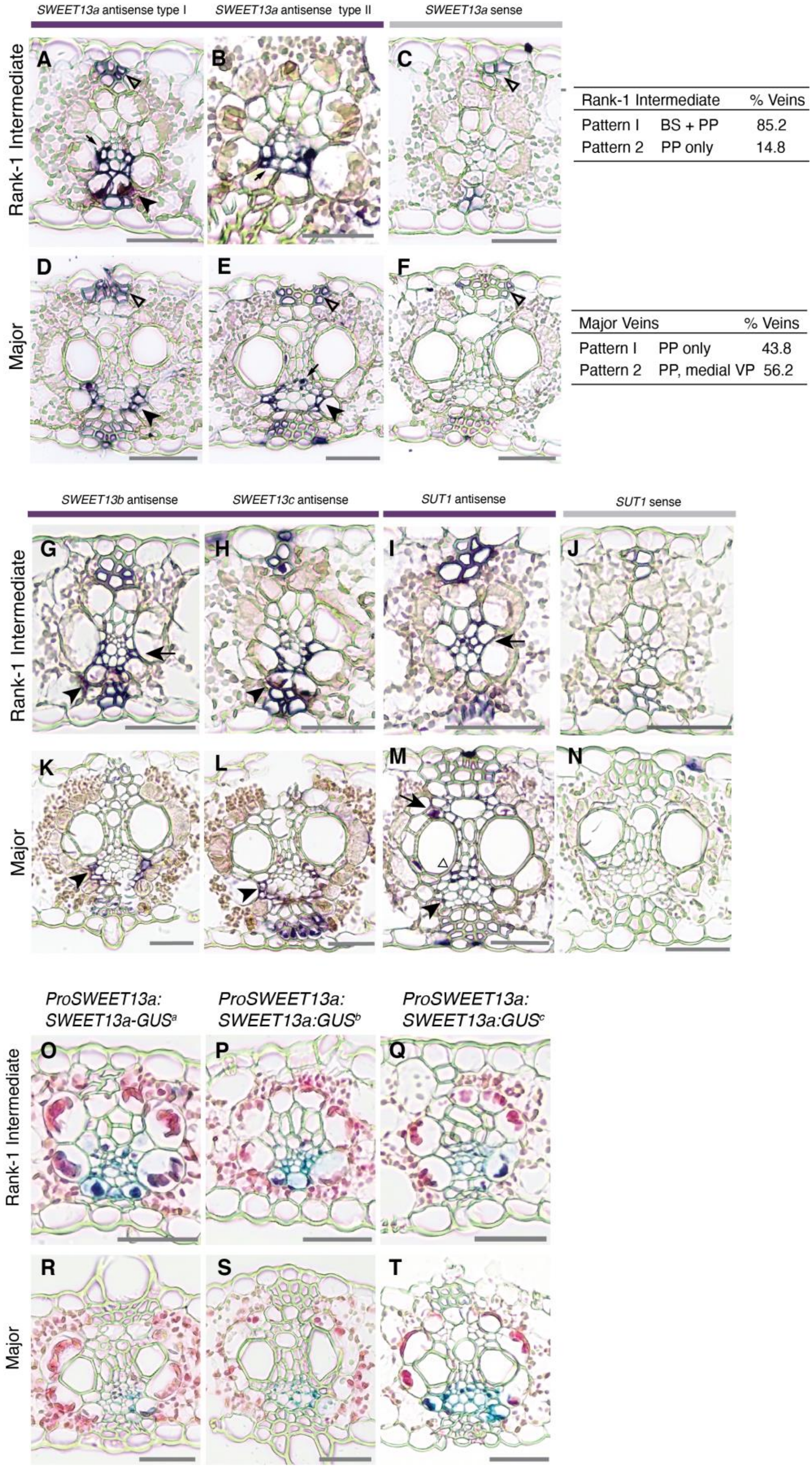

Rank-1 Intermediate		% Veins
Pattern I	BS + PP	85.2
Pattern 2	PP only	14.8

Major Veins		% Veins
Pattern I	PP only	43.8
Pattern 2	PP, medial VP	56.2

Supplemental Figure 7. SWEET and SUT mRNA Localization and SWEET13a Protein Localization in Rank-2 Intermediate and Major Veins (Supports Figure 2 and 4).

(A) to **(C)** SWEET13a mRNA localization in rank-1 intermediate veins Type I and type II refer to different patterns of staining observed using the probes **(A)** Pattern 1: ^{ab}BSC (arrowhead) and vascular parenchyma (VP) (arrow), and **(B)** Pattern 2: mainly in VP (arrow). **(C)** rank-1 vein in sense-probe-hybridized section. **(D)** to **(F)** SWEET13a mRNA localization in major veins **(D)** Pattern 1: in phloem parenchyma (PP) only (arrow), and **(E)** Pattern 2: in PP and medial VP (parenchyma between xylem and phloem) (arrow). **(F)** *SWEET13a* major vein in sense-probe - hybridized section. Most hypodermal sclerenchyma cells showed a purple dye precipitate with both sense and antisense probes, and was thus considered an artifact (open triangles). Table indicates percentage of veins meeting the above criteria for each vein type. Rank-1 intermediate, n = 196; and major veins, n = 98; variable n-numbers are due to relative proportions of each vein type in the leaf. Scale bars are 100 µm.

(G) to **(J)** *SWEET13b*, *c*, and *SUT1* mRNA localization in rank-1 intermediate veins. **(G)** For *SWEET13b* antisense probe-hybridized rank-1 intermediate veins, staining was in ^{ab}BS (arrowhead) and vascular parenchyma (arrow). **(H)** For *SWEET13c* antisense probe-hybridized rank-1 intermediate veins, staining was in ^{ab}BS (arrowhead) and vascular parenchyma (arrow). **(I)** For *SUT1* antisense probe-hybridized rank-1 intermediate veins, staining was in vasculature (arrow). **(J)** *SUT1* sense probe-hybridized rank-1 intermediate vein.

(K) to **(N)** *SWEET13b*, *c*, and *SUT1* mRNA localization in major veins. **(K)** For *SWEET13b* antisense probe-hybridized major veins, staining was in phloem parenchyma (arrowhead). **(L)** For *SWEET13c* antisense probe-hybridized major veins, staining was in phloem parenchyma (arrowhead). **(M)** For *SUT1* antisense probe-hybridized major veins, staining was in companion cells (arrow), in vascular parenchyma between xylem and phloem (triangle), and in xylem parenchyma (arrowhead). **(N)** *SUT1* sense probe-hybridized major vein. Scale bars are 100 µm.

(O) to **(T)** SWEET13a protein localization as visualized by GUS staining of SWEET13a:GUS-transformed B104 plants. Chloro-bromo-indigo precipitate is localized to abaxial portion of veins in both rank-1 intermediate (**O**) to (**Q**) and major veins **(R)** to (**T**) of all three independent transformation events. Scale bars are 100 µm; sections are counterstained with Eosin-Y.

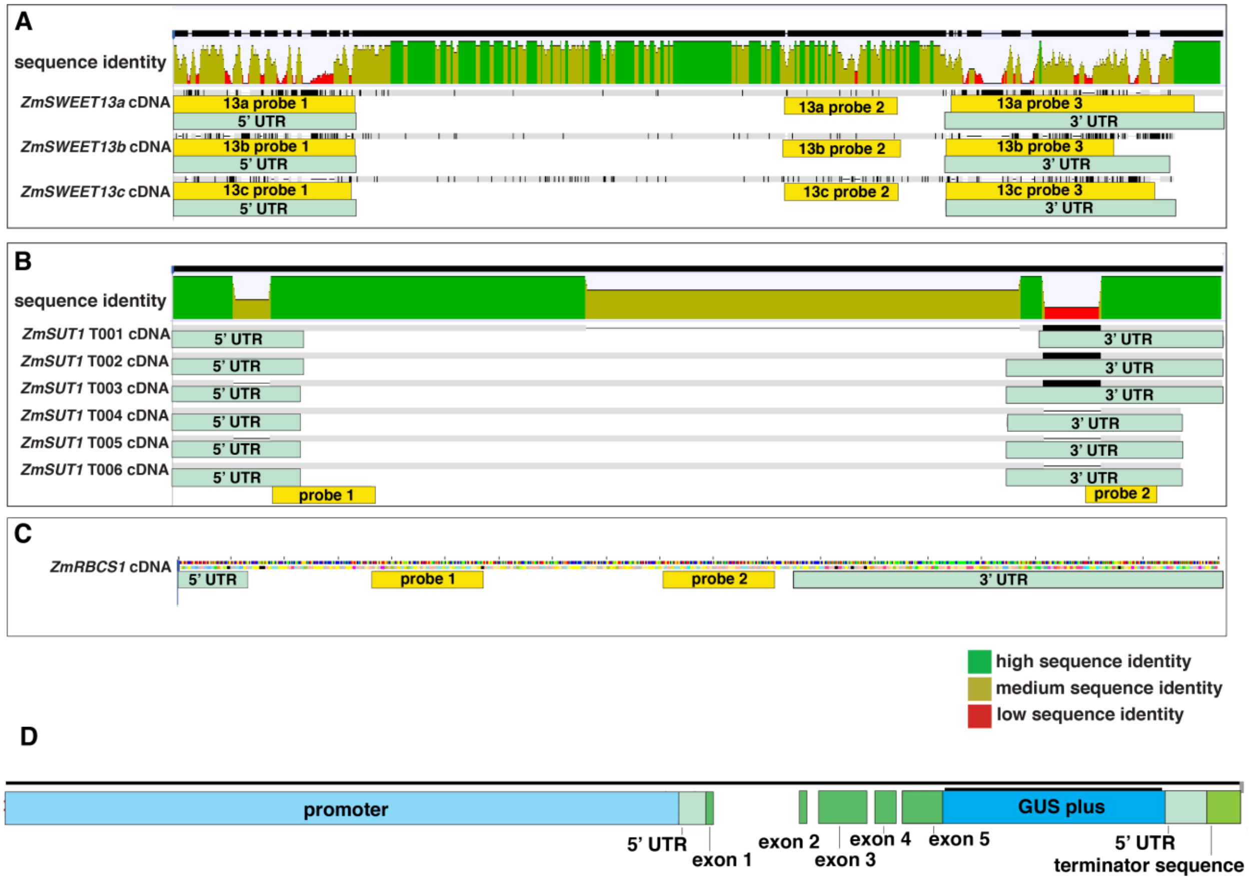

Supplemental Figure 8. Probe Design for *In Situ* Hybridization and ProSWEET13a:SWEET13a-GUS Construct Schematic (Supports Figure 2 and 4).

(A) *ZmSWEET13a*, *ZmSWEET13b*, and *ZmSWEET13c* aligned using MUSCLE in Geneious. Regions used as templates for RNA probes are highlighted in purple. Three probes in unique regions of each gene allowed us to differentiate between the homologs. **(B)** The six isoforms of *ZmSUT1* aligned using MUSCLE in Geneious; two regions common to all six isoforms were selected for probe templates. **(C)** Regions of *RBCS1* used as templates for RNA probes are highlighted in purple. **(D)** Linear schematic of *ProSWEET13a:SWEET13a-GUS* plasmid used to transform B104. The construct included the 5751 bp upstream of the start codon (light blue), all exons and introns of the SWEET13a gene (green), a 9-alanine linker fused to GUSplus (dark blue), followed by 684 bp downstream of stop codon (bright green).

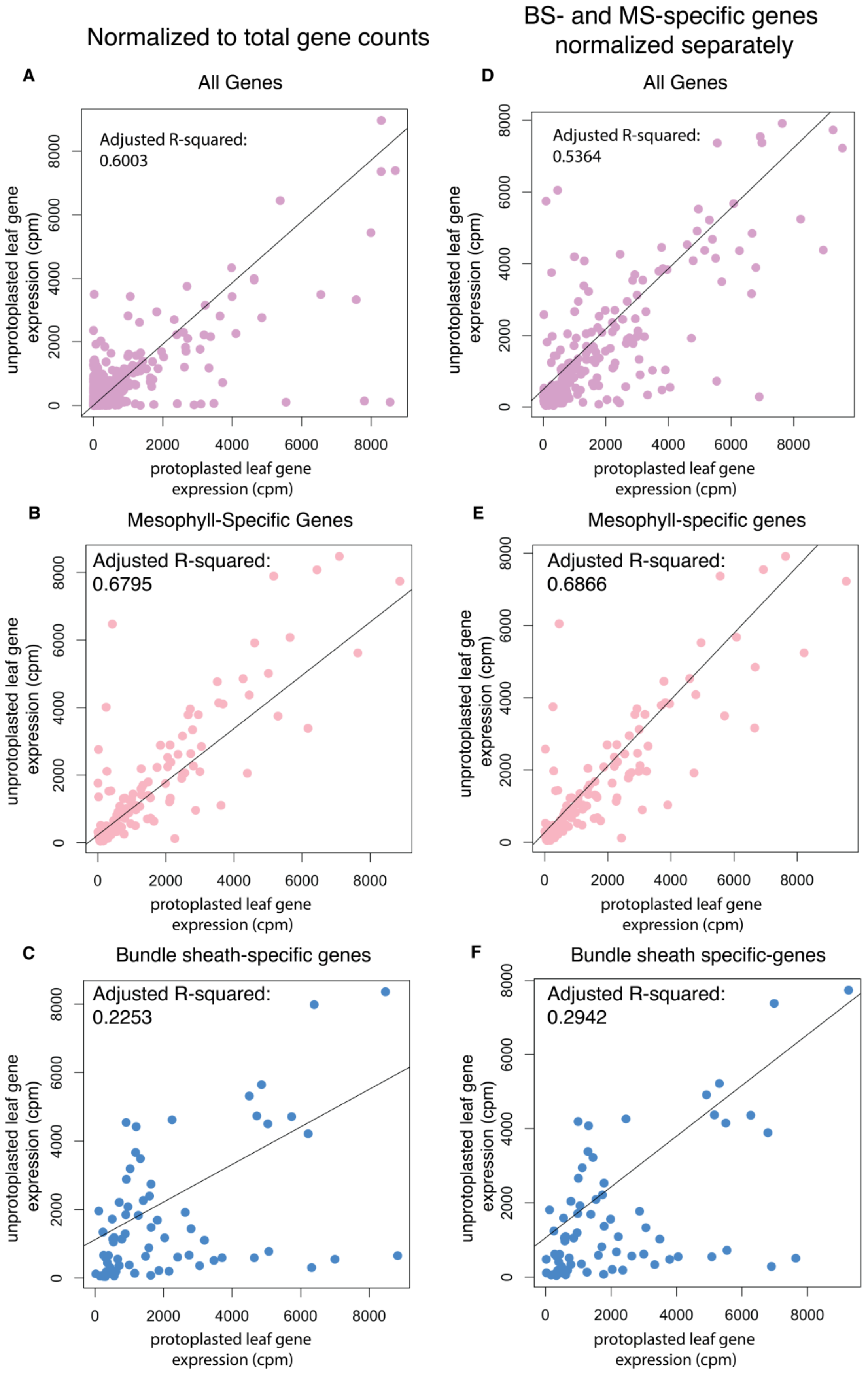
Normalized to total gene counts
BS- and MS-specific genes normalized separately
A
All Genes
Adjusted R-squared:
0.6003
D
All Genes
Adjusted R-squared:
0.5364
B
Mesophyll-Specific Genes
Adjusted R-squared:
0.6795
E
Mesophyll-specific genes
Adjusted R-squared:
0.6866
C
Bundle sheath-specific genes
Adjusted R-squared:
0.2253
F
Bundle sheath specific-genes
Adjusted R-squared:
0.2942
unprotoplasted leaf gene expression (cpm)
protoplasted leaf gene expression (cpm)
0
2000
4000
6000
8000

Supplemental Figure 9. Correlation of mRNA Counts Between Protoplasted Cells and Whole Leaf (Supports Figure 1).

(A) mRNA counts in whole leaf and protoplasted leaf expressed as counts per million reads (cpm) with adjusted R-squared values. **(B)** mRNA counts for genes enriched in mesophyll (henceforth 'MS-specific'), as determined by unsupervised marker gene discovery (see materials and methods). **(C)** mRNA counts for genes enriched in bundle sheath (henceforth: 'BS-specific'). In **(D)** to **(F)** counts of MS- and BS-specific genes were normalized separately to compensate for differing ratios of cell types represented in bulk leaf and protoplast samples. **(D)** MS- and BS-specific genes, normalized separately. **(E)** MS-specific genes normalized to total counts of MS-specific genes. **(F)** BS-specific genes normalized to total counts of BS-specific genes. TMM method of normalization.

Supplemental Table 1. mRNA Enrichment of C_4 Photosynthesis-Related Genes in MS and BS Clusters

**Mesophyll**	**Symbol or abbreviation**	**Log FC**	**Gene name**
Zm00001d031899	*MDH6*	3.00	NADP-dependent malate dehydrogenase 6
Zm00001d046170	*PEP1*	3.20	Phospho*enol*pyruvate carboxylase 1
Zm00001d044099	*CAH1*	3.58	Carbonic anhydrase 1
Zm00001d038163	*PDK1*	1.45	Pyruvate, phosphate dikinase 1
Zm00001d006520	*PDRP1*	2.95	Pyruvate, phosphate dikinase regulatory protein 1
Zm00001d035737	*GLYK*	2.19	D-glycerate 3-kinase chloroplastic
**Bundle sheath**	**Symbol or abbreviation**	**Log FC**	**Gene name**
Zm00001d004894	*RBCS2*	5.19	Ribulose biphosphate carboxylase small subunit 2
Zm00001d052595	*RBCS1*	5.18	Ribulose biphosphate carboxylase small subunit 1
Zm00001d006402	*RBCL*	2.08	Rubisco large chain (genomic, introns)
Zm00001d028471	*PCK1*	4.23	Phospho*enol*pyruvate carboxykinase 1
Zm00001d000316	*ME1*	5.04	NADP-dependent malic enzyme
Zm00001d045451	*TK1*	2.20	Transketolase 1
Zm00001d000164	*PRK2*	1.14	Phosphoribulokinase chloroplastic 4
Zm00001d048593	*RCA2*	3.79	Rubisco activase 2
Zm00001d023559	*FBPA*	5.31	Fructose-bisphosphate aldolase
Zm00001d053015	*FBPA*	2.89	Fructose-bisphosphate aldolase
Zm00001d042840	*SHBP1*	3.78	Sedoheptulose-17-bisphosphatase 3 chloroplastic
Zm00001d034241	*MDH*	1.61	Malate dehydrogenase

Supplemental Table 1: mRNA Enrichment of C_4 Photosynthesis-Related Genes in MS and BS Clusters.
For all proteins displayed in the schematic in Supplemental Figure 3A based on (Schlüter and Weber, 2020; Friso et al., 2010), mRNA expression in scRNA-seq dataset A was enriched in either mesophyll or bundle sheath cells (logFC > 1.0; FDR-adjusted p-value < .05). Gene IDs, symbols, and full names are shown along with Log FC values.

Supplemental Table 2. Genes Referenced in this Study

B73 RefGen_v4	Gene	Full name
Zm00001d023677	SWEET13a	SWEET13a
Zm00001d023673	SWEET3b	SWEET13b
Zm00001d041067	SWEET13c	SWEET13c
Zm00001d027854	SUT1	Sucrose Transporter 1
Zm00001d048611	MT1b	Metallothionein-like protein 1B
Zm00001d038558	CC3	Cystatin 3
Zm00001d004894	RBCS2	Ribulose bisphosphate carboxylase small subunit2
Zm00001d052595	RBCS1	Ribulose bisphosphate carboxylase small subunit1
Zm00001d000316	ME1	NADP-dependent malic enzyme
Zm00001d053281	NAAT1	Nicotianamine aminotransferase
Zm00001d016441	TAAT	Tyrosine aminotransferase
Zm00001d044099	CAH1	Carbonic anhydrase 1
Zm00001d031899	MD6	NADP-dependent malate dehydrogenase 6
Zm00001d046170	PEP1	Phospho*enol*pyruvate carboxylase 1
Zm00001d028471	PCK1	Phospho*enol*pyruvate carboxykinase 1
Zm00001d047658	NAS10	Nicotianamine synthase10
Zm00001d047651	NAS1	Nicotianamine synthase 1
Zm00001d028887	NAS9	Nicotianamine synthase 9
Zm00001d028888	NAS2	Nicotianamine synthase2
Zm00001d033496	Nas3	Nicotianamine synthase 3
Zm00001d035243	AAAP45	Amino acid permease 45
Zm00001d012231	AAAP56	Amino acid permease 56
Zm00001d035717	UmamiT21a	UmamiT21a
Zm00001d044951	UmamiT20a	UmamiT20a
Zm00001d038163	PDK1	Pyruvate orthophosphate dikinase1
Zm00001d031899	MDH6	NADP-dependent malate dehydrogenase 6
Zm00001d017958	GLN4	Glutamine synthetase 4 (gln1-3)
Zm00001d033747	GLN2	Glutamine synthetase 2 / Glutamine synthetase root
Zm00001d026501	GLN1	Glutamine synthetase 1 leaf isozyme chloroplastic
Zm00001d028260	GLN3	Glutamine synthetase 3 / 6 / root isozyme 5 (gln1-4)
Zm00001d044768	NRT1	Protein NRT1/ PTR FAMILY 5.8
Zm00001d027268	STP3	Sugar transport protein 3
Zm00001d019062	H+ATPase	Membrane H(+)-ATPase3
Zm00001d018178	bZIP4	Basic leucine zipper 4 / ABA-insensitive 5-like protein
Zm00001d010201	MYB25	Transcription repressor MYB6 / myb25
Zm00001d010159	ACT1	Actin 1
ENSRNA049479027	18S	Eukaryotic small subunit ribosomal RNA
AT3G48740	At-SWEET11	At-SWEET11
AT5G23660	At-SWEET12	At-SWEET12
AT5G50800	At-SWEET13	At-SWEET13
AT5G64700	At-UmamiT21	At-UmamiT21
AT4G08290	At-UmamiT20	At-UmamiT20
AT5G14940	At-At-NPF5.8	At-NPF5.8
AT3G01350	At-NPF5.9	At-NPF5.9

Supplemental Table 2. Genes Referenced in this Study.
Gene ID's are from the B73 RefGen_v4, INSDC Assembly, which is the most up-to-date B73 genome assembly currently available in the Ensembl genome browser, or Arabidopsis TAIR10 (At-). Abbreviations are used in figures throughout this manuscript, and are based on either the gene name most commonly cited in the literature, or the abbreviation found in Ensembl.

Supplemental text

Supplemental text 1. A Subset of Mesophyll Cells Specialized in Iron Metabolism Appears to be an Artifact (Supports Figure 1).

Subcluster MS5 (visible in the lower left corner of Figure 1B) shared mesophyll identity but appeared be specialized in metal accumulation and transport, as indicated by high and specific expression of four Nicotianamine synthase genes, *NAS1, 2,* 9, and *10*, which are involved in iron chelation; one iron phytosiderophore transporter. *YS1* (*yellow stripe 1*); and various additional genes involved in metal transport and metabolism (Supplemental Data 1). Initially, we hypothesized that the MS5 cell cluster could either represent a cell type with a specific localization in the leaf or may correspond to cells that contain different levels of iron. However, MS5 was missing from the dataset B, as no unique cell population was detectable which contained high levels of mRNA for multiple *Nicotianamine synthase* paralogs. The UMAP plot of the integrated dataset (Supplemental Figure 2) did not show MS5 as a well-separated cluster. Because the presence of this cluster was not reproducible, it was likely artifact of the specific growth conditions in dataset A and was not analyzed further.
