# Supplementary material for: Evidence for phloem loading via the abaxial bundle sheath cells in maize leaves
Source: Plant Cell. 2021 Jan 7;33(3):531–47. doi: 10.1093/plcell/koaa055 (PMC8136869; doi:10.1093/plcell/koaa055)
Supplement: koaa055_Supplementary_Data [file koaa055_supplementary_data.zip › tpc.00733.2020-s03.rtf]

Supplemental Data. Bezrutczyk et al. (2021). Plant Cell. Evidence for Phloem  Loading via the Abaxial Bundle Sheath Cells in Maize Leaves.

Supplemental Data Set 3. Raw Data for Phylogenetic Trees

#NEXUS
begin taxa;
	dimensions ntax=138;
	taxlabels
	AT1G01070_AtUmamiT28
	AT1G09380_AtUmamiT25
	AT1G11450_AtUmamiT27
	AT1G11460_AtUmamiT26
	AT1G21890_AtUmamiT19
	AT1G25270_AtUmamiT24
	AT1G43650_AtUmamiT22[&!color=#0080ff]
	AT1G44800_AtUmamiT18
	AT1G60050_AtUmamiT35
	AT1G68170_AtUmamiT23
	AT1G70260_AtUmamiT36
	AT1G75500_AtUmamiT05
	AT2G37450_AtUmamiT13
	AT2G37460_AtUmamiT12
	AT2G39510_AtUmamiT14
	AT2G40900_AtUmamiT11
	AT3G18200_AtUmamiT04
	AT3G28050_AtUmamiT41
	AT3G28070_AtUmamiT46
	AT3G28080_AtUmamiT47
	'AT3G28100_AT3G28100.1'
	AT3G28100_AtUmamiT45
	AT3G28130_AtUmamiT44
	AT3G30340_AtUmamiT32
	AT3G45870_AtUmamiT03
	AT3G53210_AtUmamiT06
	AT3G56620_AtUmamiT10
	AT4G01430_AtUmamiT29
	AT4G01440_AtUmamiT31
	AT4G01450_AtUmamiT30
	AT4G08290_AtUmamiT20
	AT4G08300_AtUmamiT16
	AT4G15540_AtUmamiT38
	AT4G16620_AtUmamiT08
	AT4G19185_AtUmamiT02
	AT4G28040_AtUmamiT33
	AT4G30420_AtUmamiT34
	AT5G07050_AtUmamiT09
	AT5G13670_AtUmamiT15
	AT5G40210_AtUmamiT42
	AT5G40230_AtUmamiT37
	AT5G40240_AtUmamiT40
	AT5G45370_AtUmamiT01
	AT5G47470_AtUmamiT07
	AT5G64700_AtUmamiT21[&!color=#0080ff]
	'HORVU0Hr1G023720_HORVU0Hr1G023720.1'
	'HORVU0Hr1G027480_HORVU0Hr1G027480.1'
	'HORVU0Hr1G030660_HORVU0Hr1G030660.1'
	'HORVU1Hr1G002750_HORVU1Hr1G002750.1'
	'HORVU1Hr1G002770_HORVU1Hr1G002770.1'
	'HORVU1Hr1G063340_HORVU1Hr1G063340.1'
	'HORVU1Hr1G068120_HORVU1Hr1G068120.1'
	'HORVU2Hr1G045480_HORVU2Hr1G045480.1'
	'HORVU2Hr1G046050_HORVU2Hr1G046050.1'
	'HORVU2Hr1G049250_HORVU2Hr1G049250.1'
	'HORVU2Hr1G074250_HORVU2Hr1G074250.1'
	'HORVU2Hr1G112160_HORVU2Hr1G112160.1'
	'HORVU2Hr1G121200_HORVU2Hr1G121200.1'
	'HORVU2Hr1G121210_HORVU2Hr1G121210.1'
	'HORVU2Hr1G121210_HORVU2Hr1G121210.9'
	'HORVU2Hr1G122970_HORVU2Hr1G122970.1'
	'HORVU3Hr1G000700_HORVU3Hr1G000700.1'
	'HORVU3Hr1G002710_HORVU3Hr1G002710.1'
	'HORVU3Hr1G040230_HORVU3Hr1G040230.1'
	'HORVU3Hr1G078710_HORVU3Hr1G078710.1'
	'HORVU3Hr1G079420_HORVU3Hr1G079420.1'
	'HORVU4Hr1G034530_HORVU4Hr1G034530.1'
	'HORVU4Hr1G070930_HORVU4Hr1G070930.1'
	'HORVU5Hr1G002470_HORVU5Hr1G002470.1'
	'HORVU5Hr1G002560_HORVU5Hr1G002560.1'
	'HORVU5Hr1G044210_HORVU5Hr1G044210.1'
	'HORVU5Hr1G064000_HORVU5Hr1G064000.1'
	'HORVU5Hr1G114000_HORVU5Hr1G114000.1'
	'HORVU6Hr1G000500_HORVU6Hr1G000500.1'
	'HORVU6Hr1G000600_HORVU6Hr1G000600.1'
	'HORVU6Hr1G050960_HORVU6Hr1G050960.1'
	'HORVU6Hr1G068730_HORVU6Hr1G068730.1'
	'HORVU6Hr1G080150_HORVU6Hr1G080150.1'
	'HORVU6Hr1G080220_HORVU6Hr1G080220.1'
	'HORVU6Hr1G080260_HORVU6Hr1G080260.1'
	'HORVU6Hr1G080270_HORVU6Hr1G080270.1'
	'HORVU7Hr1G002200_HORVU7Hr1G002200.1'
	'HORVU7Hr1G002600_HORVU7Hr1G002600.1'
	'HORVU7Hr1G019700_HORVU7Hr1G019700.1'
	'HORVU7Hr1G023000_HORVU7Hr1G023000.1'
	'HORVU7Hr1G023010_HORVU7Hr1G023010.1'
	'HORVU7Hr1G071230_HORVU7Hr1G071230.1'
	'HORVU7Hr1G095750_HORVU7Hr1G095750.1'
	'HORVU7Hr1G108550_HORVU7Hr1G108550.1'
	'HORVU7Hr1G109820_HORVU7Hr1G109820.1'
	Zm00001d002888_Zm00001d002888_T001
	Zm00001d002893_Zm00001d002893_T001
	Zm00001d003716_Zm00001d003716_T001
	Zm00001d007263_Zm00001d007263_T001
	Zm00001d009063_Zm00001d009063_T001
	Zm00001d009372_Zm00001d009372_T001
	Zm00001d010287_Zm00001d010287_T001
	Zm00001d010655_Zm00001d010655_T001
	Zm00001d011083_Zm00001d011083_T001
	Zm00001d011088_Zm00001d011088_T001
	Zm00001d014933_Zm00001d014933_T001
	Zm00001d017765_Zm00001d017765_T001
	Zm00001d018195_Zm00001d018195_T001
	Zm00001d020555_Zm00001d020555_T001
	Zm00001d020556_Zm00001d020556_T001
	Zm00001d021655_Zm00001d021655_T001
	Zm00001d021656_Zm00001d021656_T001
	Zm00001d025418_Zm00001d025418_T001
	Zm00001d027682_Zm00001d027682_T001
	Zm00001d029383_Zm00001d029383_T001
	Zm00001d029545_Zm00001d029545_T001
	Zm00001d030424_Zm00001d030424_T001
	Zm00001d030507_Zm00001d030507_T001
	Zm00001d031913_Zm00001d031913_T001
	Zm00001d032604_Zm00001d032604_T001
	Zm00001d033284_Zm00001d033284_T001
	Zm00001d035713_Zm00001d035713_T001
	Zm00001d035714_Zm00001d035714_T001
	Zm00001d035717_Zm00001d035717_T001[&!color=#0080ff]
	Zm00001d036123_Zm00001d036123_T001
	Zm00001d036440_Zm00001d036440_T001
	Zm00001d037160_Zm00001d037160_T001
	Zm00001d038554_Zm00001d038554_T001
	Zm00001d040089_Zm00001d040089_T001
	Zm00001d040593_Zm00001d040593_T001
	Zm00001d043171_Zm00001d043171_T001
	Zm00001d044535_Zm00001d044535_T001
	Zm00001d044951_Zm00001d044951_T001
	Zm00001d044951_Zm00001d044951_T002
	Zm00001d045567_Zm00001d045567_T001
	Zm00001d045572_Zm00001d045572_T001
	Zm00001d045576_Zm00001d045576_T001
	Zm00001d045582_Zm00001d045582_T001
	Zm00001d047208_Zm00001d047208_T001
	Zm00001d049916_Zm00001d049916_T001
	Zm00001d054055_Zm00001d054055_T001
	Zm00001d054081_Zm00001d054081_T001
	Zm00001d054085_Zm00001d054085_T001
;
end;

begin trees;
	tree tree_1 = [&R] (AT3G28070_AtUmamiT46[&!rotate=true]:0.069638,((((((((((((AT3G18200_AtUmamiT04[&!rotate=true]:0.42642,((Zm00001d040089_Zm00001d040089_T001[&!rotate=true]:0.085552,'HORVU3Hr1G002710_HORVU3Hr1G002710.1'[&!rotate=true]:0.132369)[&"bootstrap / SH-aLRT"="100/99",!rotate=true]:0.226687,Zm00001d036123_Zm00001d036123_T001[&!rotate=true]:0.482186)[&"bootstrap / SH-aLRT"="28.3/70",!rotate=true]:0.07207)[&"bootstrap / SH-aLRT"="73.6/72",!rotate=true]:0.081635,((AT1G75500_AtUmamiT05[&!rotate=true]:0.190817,((Zm00001d031913_Zm00001d031913_T001[&!rotate=true]:0.077932,Zm00001d049916_Zm00001d049916_T001[&!rotate=true]:0.054863)[&"bootstrap / SH-aLRT"="100/100",!rotate=true]:0.148621,Zm00001d054055_Zm00001d054055_T001[&!rotate=true]:0.239497)[&"bootstrap / SH-aLRT"="35.2/47",!rotate=true]:0.059146)[&"bootstrap / SH-aLRT"="94.9/100",!rotate=true]:0.08103,AT3G53210_AtUmamiT06[&!rotate=true]:0.468967)[&"bootstrap / SH-aLRT"="98.6/100",!rotate=true]:0.115202)[&"bootstrap / SH-aLRT"="98.8/96",!rotate=true]:0.193866,(((AT3G45870_AtUmamiT03[&!rotate=true]:0.2402,(AT4G19185_AtUmamiT02[&!rotate=true]:0.173754,AT5G45370_AtUmamiT01[&!rotate=true]:0.216028)[&"bootstrap / SH-aLRT"="91.8/100",!rotate=true]:0.128341)[&"bootstrap / SH-aLRT"="98.8/100",!rotate=true]:0.168978,(Zm00001d017765_Zm00001d017765_T001[&!rotate=true]:0.291194,'HORVU6Hr1G068730_HORVU6Hr1G068730.1'[&!rotate=true]:0.205413)[&"bootstrap / SH-aLRT"="91.3/100",!rotate=true]:0.191465)[&"bootstrap / SH-aLRT"="93/99",!rotate=true]:0.113861,((Zm00001d029383_Zm00001d029383_T001[&!rotate=true]:0.440815,Zm00001d047208_Zm00001d047208_T001[&!rotate=true]:0.287842)[&"bootstrap / SH-aLRT"="90.3/99",!rotate=true]:0.082325,((Zm00001d036440_Zm00001d036440_T001[&!rotate=true]:0.017003,'HORVU7Hr1G095750_HORVU7Hr1G095750.1'[&!rotate=true]:1.458149)[&"bootstrap / SH-aLRT"="99.5/99",!rotate=true]:0.175203,'HORVU6Hr1G050960_HORVU6Hr1G050960.1'[&!rotate=true]:0.152384)[&"bootstrap / SH-aLRT"="81.2/98",!rotate=true]:0.088503)[&"bootstrap / SH-aLRT"="99.7/100",!rotate=true]:0.228207)[&"bootstrap / SH-aLRT"="100/100",!rotate=true]:0.548673)[&"bootstrap / SH-aLRT"="99.4/94",!rotate=true]:0.21413,(AT4G30420_AtUmamiT34[&!rotate=true]:0.542754,(AT4G28040_AtUmamiT33[&!rotate=true]:0.522251,((Zm00001d020556_Zm00001d020556_T001[&!rotate=true]:0.214295,'HORVU5Hr1G064000_HORVU5Hr1G064000.1'[&!rotate=true]:0.384486)[&"bootstrap / SH-aLRT"="99/100",!rotate=true]:0.194646,Zm00001d020555_Zm00001d020555_T001[&!rotate=true]:0.549099)[&"bootstrap / SH-aLRT"="98.2/99",!rotate=true]:0.192618)[&"bootstrap / SH-aLRT"="28.5/31",!rotate=true]:0.092303)[&"bootstrap / SH-aLRT"="100/99",!rotate=true]:0.313236)[&"bootstrap / SH-aLRT"="12/27",!rotate=true]:0.066486,((((((AT2G40900_AtUmamiT11[&!rotate=false]:0.181538,AT3G56620_AtUmamiT10[&!rotate=false]:0.255195)[&"bootstrap / SH-aLRT"="100/100",!rotate=false]:0.279746,(AT5G07050_AtUmamiT09[&!rotate=false]:0.341773,((((Zm00001d010655_Zm00001d010655_T001[&!rotate=false]:0.097511,Zm00001d038554_Zm00001d038554_T001[&!rotate=false]:0.109425)[&"bootstrap / SH-aLRT"="99.2/100",!rotate=false]:0.096475,('HORVU0Hr1G023720_HORVU0Hr1G023720.1'[&!rotate=false]:0.005059,'HORVU1Hr1G068120_HORVU1Hr1G068120.1'[&!rotate=false]:3.0E-6)[&"bootstrap / SH-aLRT"="100/100",!rotate=false]:0.159977)[&"bootstrap / SH-aLRT"="93.5/100",!rotate=false]:0.068084,(Zm00001d043171_Zm00001d043171_T001[&!rotate=false]:0.089342,('HORVU5Hr1G044210_HORVU5Hr1G044210.1'[&!rotate=false]:0.002943,'HORVU3Hr1G078710_HORVU3Hr1G078710.1'[&!rotate=false]:2.0E-6)[&"bootstrap / SH-aLRT"="100/100",!rotate=false]:0.37664)[&"bootstrap / SH-aLRT"="92.4/100",!rotate=false]:0.052286)[&"bootstrap / SH-aLRT"="98.6/100",!rotate=false]:0.103889,(Zm00001d010287_Zm00001d010287_T001[&!rotate=false]:0.241325,'HORVU1Hr1G063340_HORVU1Hr1G063340.1'[&!rotate=false]:0.241941)[&"bootstrap / SH-aLRT"="91.7/100",!rotate=false]:0.068217)[&"bootstrap / SH-aLRT"="98.4/100",!rotate=false]:0.120273)[&"bootstrap / SH-aLRT"="97.4/100",!rotate=false]:0.1117)[&"bootstrap / SH-aLRT"="99.1/100",!rotate=false]:0.120368,(((AT1G21890_AtUmamiT19[&!rotate=false]:0.314098,(AT1G44800_AtUmamiT18[&!rotate=false]:0.187259,AT4G08300_AtUmamiT16[&!rotate=false]:0.167552)[&"bootstrap / SH-aLRT"="100/100",!rotate=false]:0.229697)[&"bootstrap / SH-aLRT"="98.3/100",!rotate=false]:0.12848,(AT4G08290_AtUmamiT20[&!rotate=false]:0.489214,(((Zm00001d018195_Zm00001d018195_T001[&!rotate=false]:0.152032,('HORVU6Hr1G080270_HORVU6Hr1G080270.1'[&!rotate=false]:0.084704,(('HORVU6Hr1G080220_HORVU6Hr1G080220.1'[&!rotate=false]:0.171876,'HORVU6Hr1G080260_HORVU6Hr1G080260.1'[&!rotate=false]:0.091939)[&"bootstrap / SH-aLRT"="97.6/100",!rotate=false]:0.093191,'HORVU6Hr1G080150_HORVU6Hr1G080150.1'[&!rotate=false]:0.168858)[&"bootstrap / SH-aLRT"="69.1/98",!rotate=false]:0.03925)[&"bootstrap / SH-aLRT"="99.6/100",!rotate=false]:0.112109)[&"bootstrap / SH-aLRT"="89.2/98",!rotate=false]:0.101747,(Zm00001d044951_Zm00001d044951_T001[&!rotate=false]:2.0E-6,Zm00001d044951_Zm00001d044951_T002[&!rotate=false]:0.028542)[&"bootstrap / SH-aLRT"="100/100",!rotate=false]:0.242926)[&"bootstrap / SH-aLRT"="100/100",!rotate=false]:0.210052,((Zm00001d030424_Zm00001d030424_T001[&!rotate=false]:3.0E-6,Zm00001d009372_Zm00001d009372_T001[&!rotate=false]:3.0E-6)[&"bootstrap / SH-aLRT"="100/100",!rotate=false]:0.295446,'HORVU7Hr1G109820_HORVU7Hr1G109820.1'[&!rotate=false]:0.14507)[&"bootstrap / SH-aLRT"="100/100",!rotate=false]:0.200459)[&"bootstrap / SH-aLRT"="96.7/100",!rotate=false]:0.116387)[&"bootstrap / SH-aLRT"="94.5/85",!rotate=false]:0.137497)[&"bootstrap / SH-aLRT"="81.7/82",!rotate=false]:0.071759,((AT2G37450_AtUmamiT13[&!rotate=false]:0.169123,AT2G37460_AtUmamiT12[&!rotate=false]:0.052349)[&"bootstrap / SH-aLRT"="100/100",!rotate=false]:0.332187,(AT2G39510_AtUmamiT14[&!rotate=false]:0.309638,(AT5G13670_AtUmamiT15[&!rotate=false]:0.460618,'HORVU2Hr1G049250_HORVU2Hr1G049250.1'[&!rotate=false]:1.876936)[&"bootstrap / SH-aLRT"="45.3/27",!rotate=false]:0.108724)[&"bootstrap / SH-aLRT"="13.1/21",!rotate=false]:0.105045)[&"bootstrap / SH-aLRT"="98.2/64",!rotate=false]:0.111925)[&"bootstrap / SH-aLRT"="84.9/68",!rotate=false]:0.083912)[&"bootstrap / SH-aLRT"="99.9/77",!rotate=false]:0.19936,(Zm00001d027682_Zm00001d027682_T001[&!rotate=false]:0.183836,Zm00001d029545_Zm00001d029545_T001[&!rotate=false]:0.34199)[&"bootstrap / SH-aLRT"="100/100",!rotate=false]:0.570153)[&"bootstrap / SH-aLRT"="54.9/54",!rotate=false]:0.082682,(AT1G43650_AtUmamiT22[&!rotate=false]:0.497571,(AT5G64700_AtUmamiT21[&!rotate=false]:0.457489,(((((((Zm00001d025418_Zm00001d025418_T001[&!rotate=false]:0.550381,Zm00001d011083_Zm00001d011083_T001[&!rotate=false]:0.228607)[&"bootstrap / SH-aLRT"="13.4/25",!rotate=false]:0.040904,((Zm00001d011088_Zm00001d011088_T001[&!rotate=false]:0.11922,Zm00001d035717_Zm00001d035717_T001[&!rotate=false]:0.101075)[&"bootstrap / SH-aLRT"="100/100",!rotate=false]:0.208922,Zm00001d035714_Zm00001d035714_T001[&!rotate=false]:0.15562)[&"bootstrap / SH-aLRT"="97/100",!rotate=false]:0.084302)[&"bootstrap / SH-aLRT"="74.8/19",!rotate=false]:0.043802,Zm00001d030507_Zm00001d030507_T001[&!rotate=false]:0.292479)[&"bootstrap / SH-aLRT"="52.4/19",!rotate=false]:0.025879,('HORVU2Hr1G112160_HORVU2Hr1G112160.1'[&!rotate=false]:0.112069,'HORVU3Hr1G079420_HORVU3Hr1G079420.1'[&!rotate=false]:0.079939)[&"bootstrap / SH-aLRT"="100/100",!rotate=false]:0.155024)[&"bootstrap / SH-aLRT"="97.5/87",!rotate=false]:0.082296,(Zm00001d035713_Zm00001d035713_T001[&!rotate=false]:0.2918,(('HORVU1Hr1G002770_HORVU1Hr1G002770.1'[&!rotate=false]:0.093814,'HORVU3Hr1G000700_HORVU3Hr1G000700.1'[&!rotate=false]:0.171063)[&"bootstrap / SH-aLRT"="94.6/100",!rotate=false]:0.063048,'HORVU1Hr1G002750_HORVU1Hr1G002750.1'[&!rotate=false]:0.169399)[&"bootstrap / SH-aLRT"="100/100",!rotate=false]:0.295353)[&"bootstrap / SH-aLRT"="90.2/92",!rotate=false]:0.064872)[&"bootstrap / SH-aLRT"="28.7/47",!rotate=false]:0.05159,Zm00001d033284_Zm00001d033284_T001[&!rotate=false]:0.41036)[&"bootstrap / SH-aLRT"="99/84",!rotate=false]:0.159913,(((Zm00001d021655_Zm00001d021655_T001[&!rotate=false]:0.125586,'HORVU2Hr1G046050_HORVU2Hr1G046050.1'[&!rotate=false]:0.72006)[&"bootstrap / SH-aLRT"="93.5/99",!rotate=false]:0.229681,(Zm00001d021656_Zm00001d021656_T001[&!rotate=false]:0.203368,'HORVU2Hr1G045480_HORVU2Hr1G045480.1'[&!rotate=false]:0.157803)[&"bootstrap / SH-aLRT"="100/100",!rotate=false]:0.320172)[&"bootstrap / SH-aLRT"="48.3/83",!rotate=false]:0.071756,((Zm00001d044535_Zm00001d044535_T001[&!rotate=false]:0.336089,'HORVU2Hr1G122970_HORVU2Hr1G122970.1'[&!rotate=false]:0.407018)[&"bootstrap / SH-aLRT"="0/77",!rotate=false]:2.0E-6,'HORVU5Hr1G114000_HORVU5Hr1G114000.1'[&!rotate=false]:0.816729)[&"bootstrap / SH-aLRT"="68/66",!rotate=false]:0.132574)[&"bootstrap / SH-aLRT"="100/72",!rotate=false]:0.247795)[&"bootstrap / SH-aLRT"="96.8/71",!rotate=false]:0.143105)[&"bootstrap / SH-aLRT"="96.3/71",!rotate=false]:0.129487)[&"bootstrap / SH-aLRT"="99.6/71",!rotate=false]:0.18779)[&"bootstrap / SH-aLRT"="38.7/51",!rotate=false]:0.047601,((((((AT1G25270_AtUmamiT24[&!rotate=false]:0.357018,AT1G68170_AtUmamiT23[&!rotate=false]:0.356857)[&"bootstrap / SH-aLRT"="92.4/100",!rotate=false]:0.306254,Zm00001d037160_Zm00001d037160_T001[&!rotate=false]:3.370242)[&"bootstrap / SH-aLRT"="3.2/19",!rotate=false]:0.052887,((Zm00001d002888_Zm00001d002888_T001[&!rotate=false]:0.275488,Zm00001d002893_Zm00001d002893_T001[&!rotate=false]:0.25476)[&"bootstrap / SH-aLRT"="99.6/100",!rotate=false]:0.221994,(Zm00001d003716_Zm00001d003716_T001[&!rotate=false]:0.210284,('HORVU2Hr1G074250_HORVU2Hr1G074250.1'[&!rotate=false]:0.070204,'HORVU4Hr1G070930_HORVU4Hr1G070930.1'[&!rotate=false]:0.130547)[&"bootstrap / SH-aLRT"="87.7/98",!rotate=false]:0.116073)[&"bootstrap / SH-aLRT"="100/99",!rotate=false]:0.424497)[&"bootstrap / SH-aLRT"="74.8/80",!rotate=false]:0.106378)[&"bootstrap / SH-aLRT"="98.5/64",!rotate=false]:0.162784,(AT1G09380_AtUmamiT25[&!rotate=false]:0.449766,(((Zm00001d009063_Zm00001d009063_T001[&!rotate=false]:0.121783,Zm00001d040593_Zm00001d040593_T001[&!rotate=false]:0.111142)[&"bootstrap / SH-aLRT"="100/100",!rotate=false]:0.23481,'HORVU3Hr1G040230_HORVU3Hr1G040230.1'[&!rotate=false]:0.235538)[&"bootstrap / SH-aLRT"="80.2/98",!rotate=false]:0.1329,'HORVU7Hr1G108550_HORVU7Hr1G108550.1'[&!rotate=false]:0.423448)[&"bootstrap / SH-aLRT"="94.5/100",!rotate=false]:0.142779)[&"bootstrap / SH-aLRT"="98.5/100",!rotate=false]:0.173465)[&"bootstrap / SH-aLRT"="92.4/66",!rotate=false]:0.102079,((Zm00001d014933_Zm00001d014933_T001[&!rotate=false]:0.367379,Zm00001d045572_Zm00001d045572_T001[&!rotate=false]:0.375484)[&"bootstrap / SH-aLRT"="98.7/100",!rotate=false]:0.23434,((Zm00001d007263_Zm00001d007263_T001[&!rotate=false]:0.272999,('HORVU7Hr1G023010_HORVU7Hr1G023010.1'[&!rotate=false]:0.060648,'HORVU7Hr1G023000_HORVU7Hr1G023000.1'[&!rotate=false]:0.053452)[&"bootstrap / SH-aLRT"="99.2/100",!rotate=false]:0.199658)[&"bootstrap / SH-aLRT"="82.1/99",!rotate=false]:0.126512,('HORVU2Hr1G121200_HORVU2Hr1G121200.1'[&!rotate=false]:0.182817,('HORVU2Hr1G121210_HORVU2Hr1G121210.1'[&!rotate=false]:0.00447,'HORVU2Hr1G121210_HORVU2Hr1G121210.9'[&!rotate=false]:0.0096)[&"bootstrap / SH-aLRT"="83.6/100",!rotate=false]:0.060127)[&"bootstrap / SH-aLRT"="99.8/100",!rotate=false]:0.372711)[&"bootstrap / SH-aLRT"="100/100",!rotate=false]:0.357796)[&"bootstrap / SH-aLRT"="100/100",!rotate=false]:0.377626)[&"bootstrap / SH-aLRT"="36.7/48",!rotate=false]:0.077304,((((((AT1G11450_AtUmamiT27[&!rotate=false]:0.078741,AT1G11460_AtUmamiT26[&!rotate=false]:0.090641)[&"bootstrap / SH-aLRT"="99.2/100",!rotate=false]:0.114334,AT1G01070_AtUmamiT28[&!rotate=false]:0.112619)[&"bootstrap / SH-aLRT"="98.2/100",!rotate=false]:0.109237,AT4G01430_AtUmamiT29[&!rotate=false]:0.195173)[&"bootstrap / SH-aLRT"="100/100",!rotate=false]:0.317762,(AT4G01450_AtUmamiT30[&!rotate=false]:0.418902,AT4G01440_AtUmamiT31[&!rotate=false]:0.23793)[&"bootstrap / SH-aLRT"="71.9/99",!rotate=false]:0.117667)[&"bootstrap / SH-aLRT"="99/100",!rotate=false]:0.178987,AT3G30340_AtUmamiT32[&!rotate=false]:0.51868)[&"bootstrap / SH-aLRT"="99.4/100",!rotate=false]:0.211021,((((Zm00001d045576_Zm00001d045576_T001[&!rotate=false]:0.148098,('HORVU7Hr1G002200_HORVU7Hr1G002200.1'[&!rotate=false]:0.528348,'HORVU7Hr1G019700_HORVU7Hr1G019700.1'[&!rotate=false]:0.145486)[&"bootstrap / SH-aLRT"="95.6/100",!rotate=false]:0.098781)[&"bootstrap / SH-aLRT"="99.1/100",!rotate=false]:0.244787,'HORVU4Hr1G034530_HORVU4Hr1G034530.1'[&!rotate=false]:0.7106)[&"bootstrap / SH-aLRT"="33.8/92",!rotate=false]:0.07144,(((Zm00001d045567_Zm00001d045567_T001[&!rotate=false]:0.145074,'HORVU7Hr1G002600_HORVU7Hr1G002600.1'[&!rotate=false]:0.09736)[&"bootstrap / SH-aLRT"="99.5/98",!rotate=false]:0.141061,Zm00001d054085_Zm00001d054085_T001[&!rotate=false]:0.235227)[&"bootstrap / SH-aLRT"="44.1/89",!rotate=false]:0.083829,'HORVU6Hr1G000500_HORVU6Hr1G000500.1'[&!rotate=false]:0.158894)[&"bootstrap / SH-aLRT"="100/98",!rotate=false]:0.250657)[&"bootstrap / SH-aLRT"="63.5/92",!rotate=false]:0.09248,(Zm00001d054081_Zm00001d054081_T001[&!rotate=false]:0.196517,(('HORVU5Hr1G002560_HORVU5Hr1G002560.1'[&!rotate=false]:0.608043,'HORVU6Hr1G000600_HORVU6Hr1G000600.1'[&!rotate=false]:0.054114)[&"bootstrap / SH-aLRT"="61.9/94",!rotate=false]:0.065512,'HORVU5Hr1G002470_HORVU5Hr1G002470.1'[&!rotate=false]:0.34037)[&"bootstrap / SH-aLRT"="99.2/100",!rotate=false]:0.156864)[&"bootstrap / SH-aLRT"="100/100",!rotate=false]:0.238137)[&"bootstrap / SH-aLRT"="99.5/100",!rotate=false]:0.191323)[&"bootstrap / SH-aLRT"="99.8/100",!rotate=false]:0.226263)[&"bootstrap / SH-aLRT"="95.3/64",!rotate=false]:0.099402)[&"bootstrap / SH-aLRT"="90.9/74",!rotate=false]:0.070608)[&"bootstrap / SH-aLRT"="73.2/67",!rotate=true]:0.106603,((AT4G16620_AtUmamiT08[&!rotate=true]:0.475661,AT5G47470_AtUmamiT07[&!rotate=true]:0.295461)[&"bootstrap / SH-aLRT"="99.3/100",!rotate=true]:0.250716,(Zm00001d045582_Zm00001d045582_T001[&!rotate=true]:0.45103,(('HORVU0Hr1G030660_HORVU0Hr1G030660.1'[&!rotate=true]:0.0,'HORVU7Hr1G071230_HORVU7Hr1G071230.1'[&!rotate=true]:0.0)[&!rotate=true]:3.0E-6,'HORVU0Hr1G027480_HORVU0Hr1G027480.1'[&!rotate=true]:3.0E-6)[&"bootstrap / SH-aLRT"="100/100",!rotate=true]:0.471881)[&"bootstrap / SH-aLRT"="99.3/100",!rotate=true]:0.279656)[&"bootstrap / SH-aLRT"="100/100",!rotate=true]:0.524236)[&"bootstrap / SH-aLRT"="91.2/86",!rotate=true]:0.263552,Zm00001d032604_Zm00001d032604_T001[&!rotate=true]:2.280458)[&"bootstrap / SH-aLRT"="0/89",!rotate=true]:0.020111,(AT1G60050_AtUmamiT35[&!rotate=true]:0.365997,AT1G70260_AtUmamiT36[&!rotate=true]:0.251061)[&"bootstrap / SH-aLRT"="100/100",!rotate=true]:0.578199)[&"bootstrap / SH-aLRT"="99.4/100",!rotate=true]:0.268223,(AT3G28050_AtUmamiT41[&!rotate=true]:0.377134,((AT5G40230_AtUmamiT37[&!rotate=true]:0.109213,AT4G15540_AtUmamiT38[&!rotate=true]:0.198952)[&"bootstrap / SH-aLRT"="84.2/100",!rotate=true]:0.07146,AT5G40240_AtUmamiT40[&!rotate=true]:0.144619)[&"bootstrap / SH-aLRT"="100/100",!rotate=true]:0.363281)[&"bootstrap / SH-aLRT"="41/54",!collapse={"collapsed",0.0},!rotate=true]:0.091561)[&"bootstrap / SH-aLRT"="98.6/100",!rotate=true]:0.184511,AT5G40210_AtUmamiT42[&!rotate=true]:0.317458)[&"bootstrap / SH-aLRT"="99.7/100",!rotate=true]:0.184502,AT3G28130_AtUmamiT44[&!rotate=true]:0.226525)[&"bootstrap / SH-aLRT"="99.7/100",!rotate=true]:0.146139,AT3G28080_AtUmamiT47[&!rotate=true]:0.077455)[&"bootstrap / SH-aLRT"="5.7/57",!rotate=true]:0.023938,(AT3G28100_AtUmamiT45[&!rotate=true]:0.025384,'AT3G28100_AT3G28100.1'[&!rotate=true]:3.0E-6)[&"bootstrap / SH-aLRT"="99.7/100",!rotate=true]:0.061223);
end;

begin figtree;
	set appearance.backgroundColorAttribute="Default";
	set appearance.backgroundColour=#ffffff;
	set appearance.branchColorAttribute="bootstrap / SH-aLRT";
	set appearance.branchColorGradient=false;
	set appearance.branchLineWidth=1.0;
	set appearance.branchMinLineWidth=0.0;
	set appearance.branchWidthAttribute="Fixed";
	set appearance.foregroundColour=#000000;
	set appearance.hilightingGradient=false;
	set appearance.selectionColour=#2d3680;
	set branchLabels.colorAttribute="User selection";
	set branchLabels.displayAttribute="bootstrap / SH-aLRT";
	set branchLabels.fontName="sansserif";
	set branchLabels.fontSize=8;
	set branchLabels.fontStyle=0;
	set branchLabels.isShown=false;
	set branchLabels.significantDigits=4;
	set colour.order.bootstrap / sh-alrt="bootstrap / SH-aLRT:0/77,0/89,100/100,100/72,100/98,100/99,12/27,13.1/21,13.4/25,28.3/70,28.5/31,28.7/47,3.2/19,33.8/92,35.2/47,36.7/48,38.7/51,41/54,44.1/89,45.3/27,48.3/83,5.7/57,52.4/19,54.9/54,61.9/94,63.5/92,68/66,69.1/98,71.9/99,73.2/67,73.6/72,74.8/19,74.8/80,80.2/98,81.2/98,81.7/82,82.1/99,83.6/100,84.2/100,84.9/68,87.7/98,89.2/98,90.2/92,90.3/99,90.9/74,91.2/86,91.3/100,91.7/100,91.8/100,92.4/100,92.4/66,93.5/100,93.5/99,93/99,94.5/100,94.5/85,94.6/100,94.9/100,95.3/64,95.6/100,96.3/71,96.7/100,96.8/71,97.4/100,97.5/87,97.6/100,97/100,98.2/100,98.2/64,98.2/99,98.3/100,98.4/100,98.5/100,98.5/64,98.6/100,98.7/100,98.8/100,98.8/96,99.1/100,99.2/100,99.3/100,99.4/100,99.4/94,99.5/100,99.5/98,99.5/99,99.6/100,99.6/71,99.7/100,99.8/100,99.9/77,99/100,99/84";
	set colour.scheme.bootstrap / sh-alrt="bootstrap / SH-aLRT:FixedDiscrete{FIXED}";
	set layout.expansion=50;
	set layout.layoutType="RECTILINEAR";
	set layout.zoom=0;
	set legend.attribute="bootstrap / SH-aLRT";
	set legend.fontSize=10.0;
	set legend.isShown=false;
	set legend.significantDigits=4;
	set nodeBars.barWidth=4.0;
	set nodeBars.displayAttribute=null;
	set nodeBars.isShown=false;
	set nodeLabels.colorAttribute="User selection";
	set nodeLabels.displayAttribute="Node ages";
	set nodeLabels.fontName="sansserif";
	set nodeLabels.fontSize=8;
	set nodeLabels.fontStyle=0;
	set nodeLabels.isShown=false;
	set nodeLabels.significantDigits=4;
	set nodeShapeExternal.colourAttribute="User selection";
	set nodeShapeExternal.isShown=false;
	set nodeShapeExternal.minSize=10.0;
	set nodeShapeExternal.scaleType=Width;
	set nodeShapeExternal.shapeType=Circle;
	set nodeShapeExternal.size=4.0;
	set nodeShapeExternal.sizeAttribute="Fixed";
	set nodeShapeInternal.colourAttribute="User selection";
	set nodeShapeInternal.isShown=false;
	set nodeShapeInternal.minSize=10.0;
	set nodeShapeInternal.scaleType=Width;
	set nodeShapeInternal.shapeType=Circle;
	set nodeShapeInternal.size=4.0;
	set nodeShapeInternal.sizeAttribute="Fixed";
	set polarLayout.alignTipLabels=false;
	set polarLayout.angularRange=0;
	set polarLayout.rootAngle=0;
	set polarLayout.rootLength=100;
	set polarLayout.showRoot=true;
	set radialLayout.spread=0.0;
	set rectilinearLayout.alignTipLabels=true;
	set rectilinearLayout.curvature=0;
	set rectilinearLayout.rootLength=0;
	set scale.offsetAge=0.0;
	set scale.rootAge=1.0;
	set scale.scaleFactor=1.0;
	set scale.scaleRoot=false;
	set scaleAxis.automaticScale=true;
	set scaleAxis.fontSize=8.0;
	set scaleAxis.isShown=false;
	set scaleAxis.lineWidth=1.0;
	set scaleAxis.majorTicks=1.0;
	set scaleAxis.minorTicks=0.5;
	set scaleAxis.origin=0.0;
	set scaleAxis.reverseAxis=false;
	set scaleAxis.showGrid=true;
	set scaleBar.automaticScale=true;
	set scaleBar.fontSize=10.0;
	set scaleBar.isShown=false;
	set scaleBar.lineWidth=1.0;
	set scaleBar.scaleRange=3.0;
	set tipLabels.colorAttribute="User selection";
	set tipLabels.displayAttribute="Names";
	set tipLabels.fontName="sansserif";
	set tipLabels.fontSize=8;
	set tipLabels.fontStyle=0;
	set tipLabels.isShown=true;
	set tipLabels.significantDigits=4;
	set trees.order=false;
	set trees.orderType="increasing";
	set trees.rooting=true;
	set trees.rootingType="User Selection";
	set trees.transform=true;
	set trees.transformType="cladogram";
end;
